# A smart “off–on” gate for the *in situ* detection of hydrogen sulphide with Cu(ii)-assisted europium emission†
†Electronic supplementary information (ESI) available: Detailed experimental procedures, characterization of compounds, NMR spectra and supplementary fluorometric titration studies. See DOI: 10.1039/c5sc04091d


**DOI:** 10.1039/c5sc04091d

**Published:** 2015-12-07

**Authors:** Zhenhao Liang, Tik-Hung Tsoi, Chi-Fai Chan, Lixiong Dai, Yudan Wu, Guangyan Du, Lizhi Zhu, Chi-Sing Lee, Wing-Tak Wong, Ga-Lai Law, Ka-Leung Wong

**Affiliations:** a Laboratory of Chemical Genomics , School of Chemical Biology and Biotechnology , Peking University Shenzhen Graduate School , Shenzhen University Town , Xili , Shenzhen 518055 , China . Email: lizc@pkusz.edu.cn; b State Key Laboratory for Chiral Sciences , Department of Applied Biological and Chemical Technology , Hong Kong Polytechnic University Shenzhen Research Institute , Shenzhen , China; c Department of Chemistry , Hong Kong Baptist University , Kowloon Tong , Hong Kong

## Abstract

A water-soluble and emissive Eu-complex (**EuL1**) bearing a DO3A(Eu^3+^)–pyridine–aza-crown motif has been prepared and its Cu^2+^ complex has been demonstrated to be a smart luminescence “off–on” gate for H_2_S detection in water with a nano-molar detection limit (60 nM). **EuL1** binds to Cu^2+^ ions selectively (*K*_B_ = 1.2 × 10^5^ M^–1^) inducing 17-fold luminescence quenching and forming a 1 : 1 stoichiometric complex (**EuL1**–Cu^2+^), which responds to H_2_S selectively with restoration of the original Eu emission of **EuL1** followed by a further 40-fold luminescence enhancement, forming a 1 : 1 stoichiometric complex (**EuL1**–Na_2_S, *K*_B_ = 1.5 × 10^4^ M^–1^). Without Cu^2+^ ions, **EuL1** showed non-specific binding towards H_2_S with only a 5-fold luminescence enhancement.

## Abstract 2

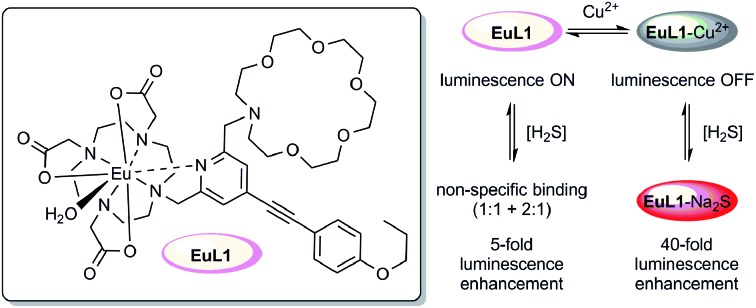
A novel responsive europium-based luminescence “off–on” gate for the *in situ* detection of H_2_S in water was developed.

## Introduction

Hydrogen sulphide (H_2_S) is the smallest bioactive thiol that may act as a gaseous signalling agent,^1^ and its production in different tissue types is associated with a wide range of physiological responses such as vascular smooth muscle relaxation,^2^ mitochondrial ATP production,^3^ insulin-signalling inhibition,^4^ regulation of inflammation response^5^ and mediation of neurotransmission.^6^ Moreover, recent investigations show that abnormal levels of H_2_S are associated with a variety of diseases, such as neurodegenerative diseases,^7^ diabetes^8^ and cancer.^9^ However, the biological targets of H_2_S and the mechanisms of these H_2_S-related physiological phenomena remain unclear. Therefore the development of responsive and reversible luminescence probes for non-invasive real time monitoring of H_2_S may be useful for understanding its biological modes of action.

One of the major approaches for developing luminescence H_2_S detection^10^ is based on sulphide-specific chemical reactions, such as reduction of an azide^11^ and nucleophilic addition of a sulphide ion.^12^ This type of luminescence probe is generally irreversible and usually requires a considerably long incubation time. An alternative approach is based on CuS precipitation^13^ due to the low-solubility of CuS (*K*_sp_ = 6.3 × 10^–36^). These luminescence probes are generally reversible with low detection limits. We are particularly interested in developing H_2_S luminescence sensors based on organo-lanthanide complexes due to their water-solubility and unique photophysical properties, including line-like emission spectra and long luminescence lifetimes (micro to milli second scale) that can effectively separate the observing signal from biological autofluorescence noise and are suitable for time-gated detection. Recently, a few studies have been found in the literature with irreversible H_2_S lanthanide probes.12*a* Herein, we report the development of a novel responsive europium-based luminescence “off–on” gate for the *in situ* detection of H_2_S in water.

**Fig. 1:**
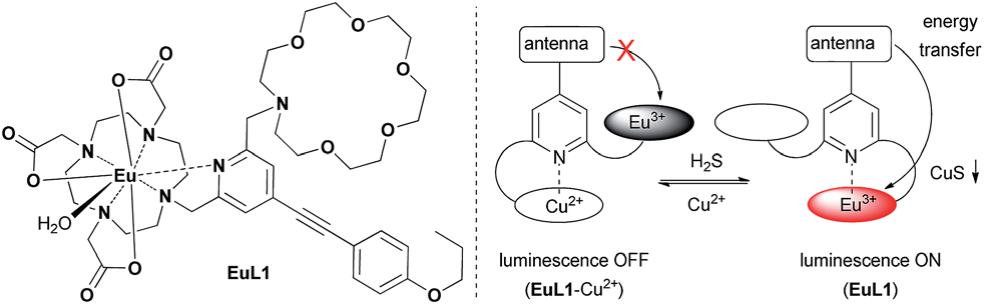
The structure of **EuL1** and the illustration of the design of a reversible Eu-based luminescence probe (**EuL1**–Cu^2+^) for H_2_S detection.

As illustrated in Fig. 1, **EuL1** contains a DO3A–Eu^3+^ complex and an aza-18-crown-6 moiety, which are linked to the 2- and 6-positions of a pyridine-containing chromophore constituting a switch-like structure. In the ground state, **EuL1** should be emissive due to the coordination of the pyridine chromophore to a Eu^3+^ ion, which favours energy transfer from the organic chromophore to the Eu^3+^ ion. Upon binding of the aza-18-crown-6 moiety with a Cu^2+^ ion, pyridine is expected to coordinate with the Cu^2+^ ion, resulting in luminescence quenching. The europium emission should be recovered after the displacement of the Cu^2+^ ion upon copper sulphide precipitation.

## Results and discussion

### Synthesis and photophysical properties of **L1** and **EuL1**

**Scheme 1:**
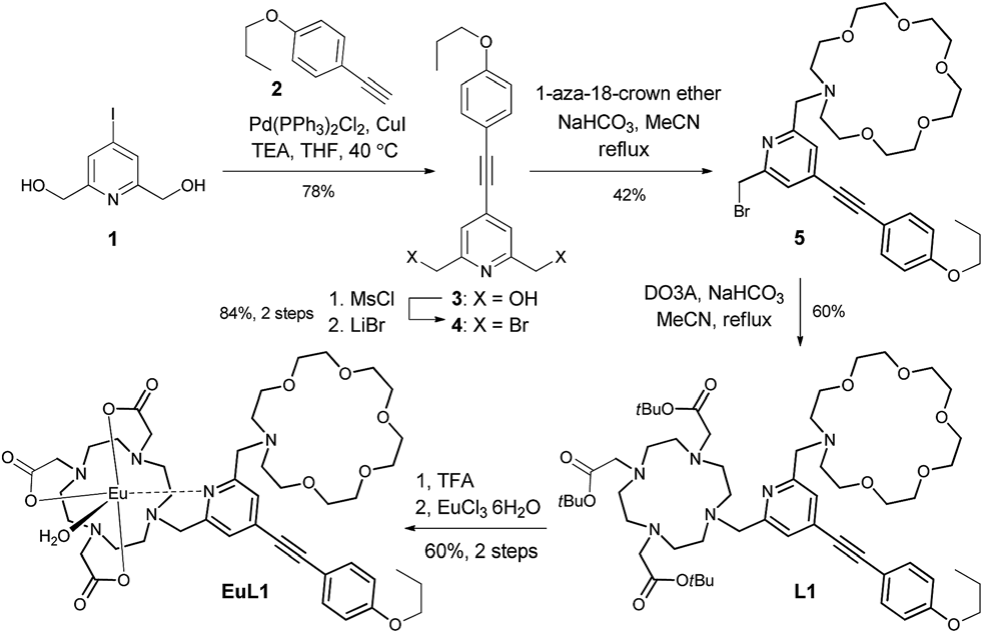
Synthesis of **L1** and **EuL1**.

Ligand **L1** was readily prepared from (4-iodopyridine-2,6-diyl)dimethanol (**1**)^14^*via* a desymmetrization synthetic strategy. As shown in Scheme 1, a pyridine-containing chromophore (based on a D–π–A motif) was established *via* a Sonogashira cross-coupling reaction between **1** and 1-ethynyl-4-propoxybenzene (**2**).^15^ After converting both hydroxyl groups of **3** into the corresponding bromide, the aza-18-crown-6 and DO3A moieties were incorporated into **4** sequentially under basic conditions and afforded **L1** in good yields. **L1** was fully characterized using ^1^H and ^13^C NMR spectroscopy and HRMS. Finally, acid hydrolysis of the *t*-butyl esters followed by Eu complex formation provided **EuL1**, which was characterized unambiguously using HRMS and HPLC (Table S1 and Fig. S1†).

**Fig. 2:**
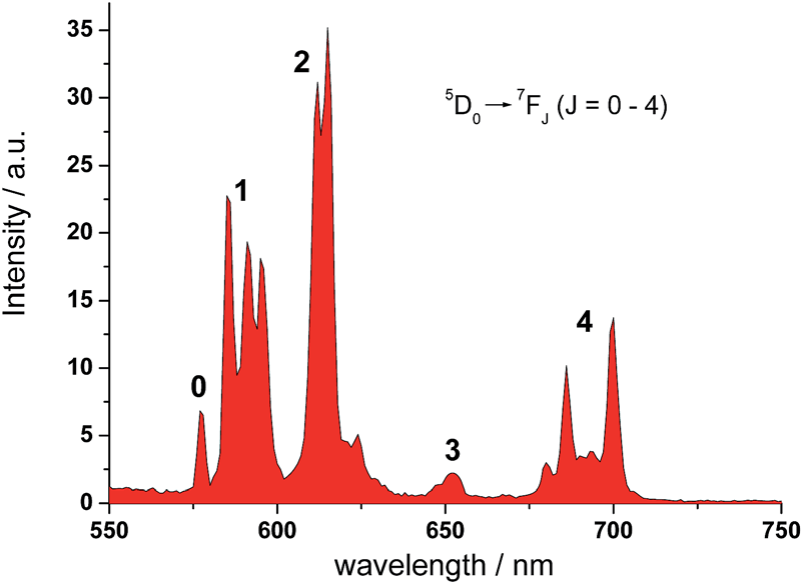
Emission spectrum of **EuL1** (H_2_O, *λ*_ex_ = 325 nm, 10 μM).

In the UV-vis absorption spectrum, **L1** showed strong absorption bands at 235 and 310 nm in methanol which are attributed to the π to π* transitions. The absorption bands were broadened and red-shifted in **EuL1** (245 and 333 nm, *ε*_333 nm_ = 7560 M^–1^ cm^–1^) in water (Fig. S2†). The excitation spectrum of **EuL1** at 615 nm showed maxima at 240 and 340 nm (Fig. S2†), evidencing an antenna effect due to energy transfer from the ligand to the Eu^3+^ ion. The ^5^D_0_ → ^7^F_*J*_ transitions of **EuL1** (*λ*_ex_ = 325 nm) were found at 578 (*J* = 0), 585–603 (*J* = 1), 604–637 (*J* = 2), 646–658 (*J* = 3), and 673–712 nm (*J* = 4) in the emission spectrum (Fig. 2). The quantum yield of **EuL1** corresponding to the ^5^D_0_ → ^7^F_2_ transitions of Eu^3+^ ions in water is 0.5% (Table S2†).

### Fluorimetric titration studies of **EuL1**

**Fig. 3:**
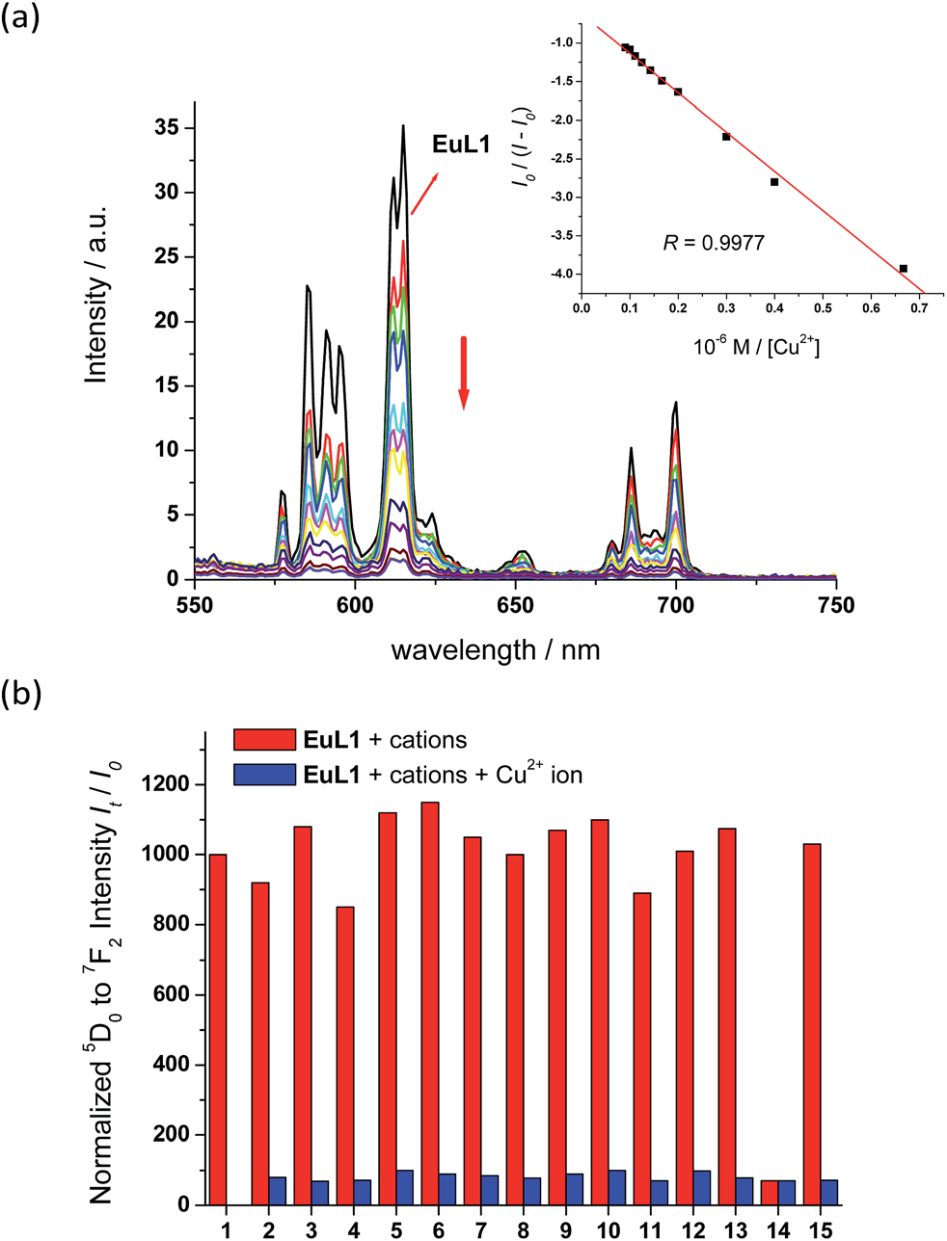
(a) Fluorimetric titration of **EuL1** (10 μM) towards Cu^2+^. The inset shows the plot of *I*_0_/(*I* – *I*_0_) *vs.* [Cu^2+^] (0–20 μM). *I* and *I*_0_ stand for intensity of europium emission ^5^D_0_ → ^7^F_2_. (b) Effects of various metal ions on the luminescence intensity of **EuL1** (10 μM). 1: **EuL1** only; 2: Na^+^; 3: K^+^; 4: Ca^2+^; 5: Mg^2+^; 6: Ba^2+^; 7: Co^2+^; 8: Zn^2+^; 9: Ni^2+^; 10: Fe^2+^; 11: Mn^2+^; 12: Cu^+^; 13: Li^+^; 14: Cu^2+^; 15: all of the above metal ions except Cu^2+^. All spectra were acquired in water with excitation at 325 nm.

With **EuL1** in hand, its binding properties towards Cu^2+^ ions were investigated. Upon the addition of 1 equiv. of Cu^2+^ ions (CuCl_2_ as the source of Cu^2+^ ions), the absorption maximum of **EuL1** showed a slight red shift and the absorption ability slightly decreased due to the effect of the copper metal. In a titration study, **EuL1** exhibited a 17-fold quenching of the europium emission with an excess of Cu^2+^ ions and the Benesi–Hildebrand plot showed a 1 : 1 binding stoichiometry with *K*_B_ = 1.2 × 10^5^ M^–1^ (inset of Fig. 3a).^16^ The Job's plot also supported the formation of a **EuL1**–Cu^2+^ complex in a 1 : 1 ratio (Fig. S3†). In a competitive study, the addition of a large excess of various metal ions, such as Na^+^, K^+^, Ca^2+^, Mg^2+^, Ba^2+^, Co^2+^, Zn^2+^, Ni^2+^, Fe^2+^, Mn^2+^, Cu^+^ and Li^+^ ions, to **EuL1** resulted in only slight luminescence changes (red columns in Fig. 3b). The subsequent addition of excess Cu^2+^ ions caused significant luminescence quenching (blue columns in Fig. 3b). These results indicate the high selectivity of **EuL1** towards Cu^2+^ ions and that the binding between **EuL1** and Cu^2+^ ions is not interfered by other metal ions. In a pH study, **EuL1** remains highly emissive and was quenched by Cu^2+^ ions in the pH range 6 to 8 (Fig. S4†), indicating that **EuL1** is stable and can bind to Cu^2+^ ions under physiological conditions.

**Fig. 4:**
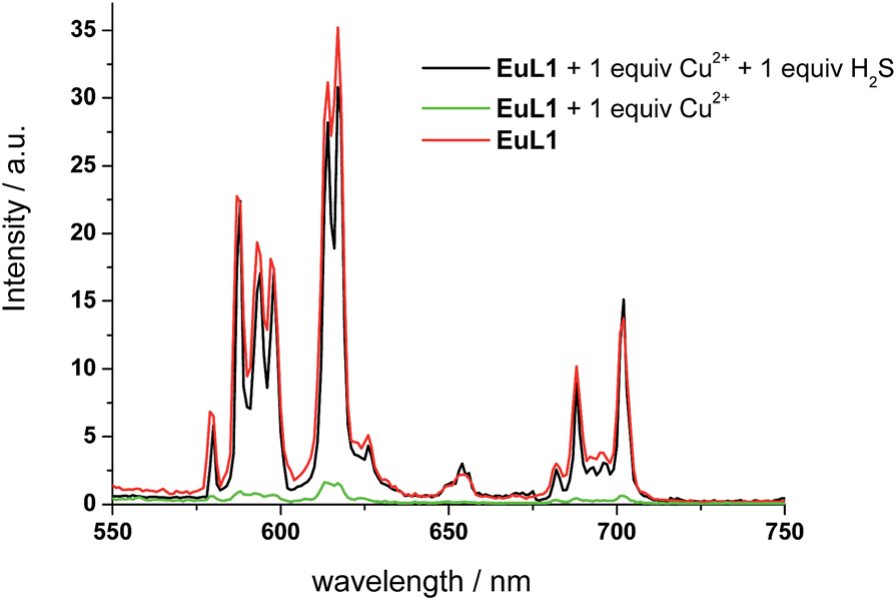
The emission spectra of **EuL1** (10 μM) (red), with 1 equiv. of Cu^2+^ ions (green), and with 1 equiv. of Cu^2+^ ions and 1 equiv. of H_2_S (black). All spectra were acquired in water with *λ*_ex_ at 325 nm.

**Fig. 5:**
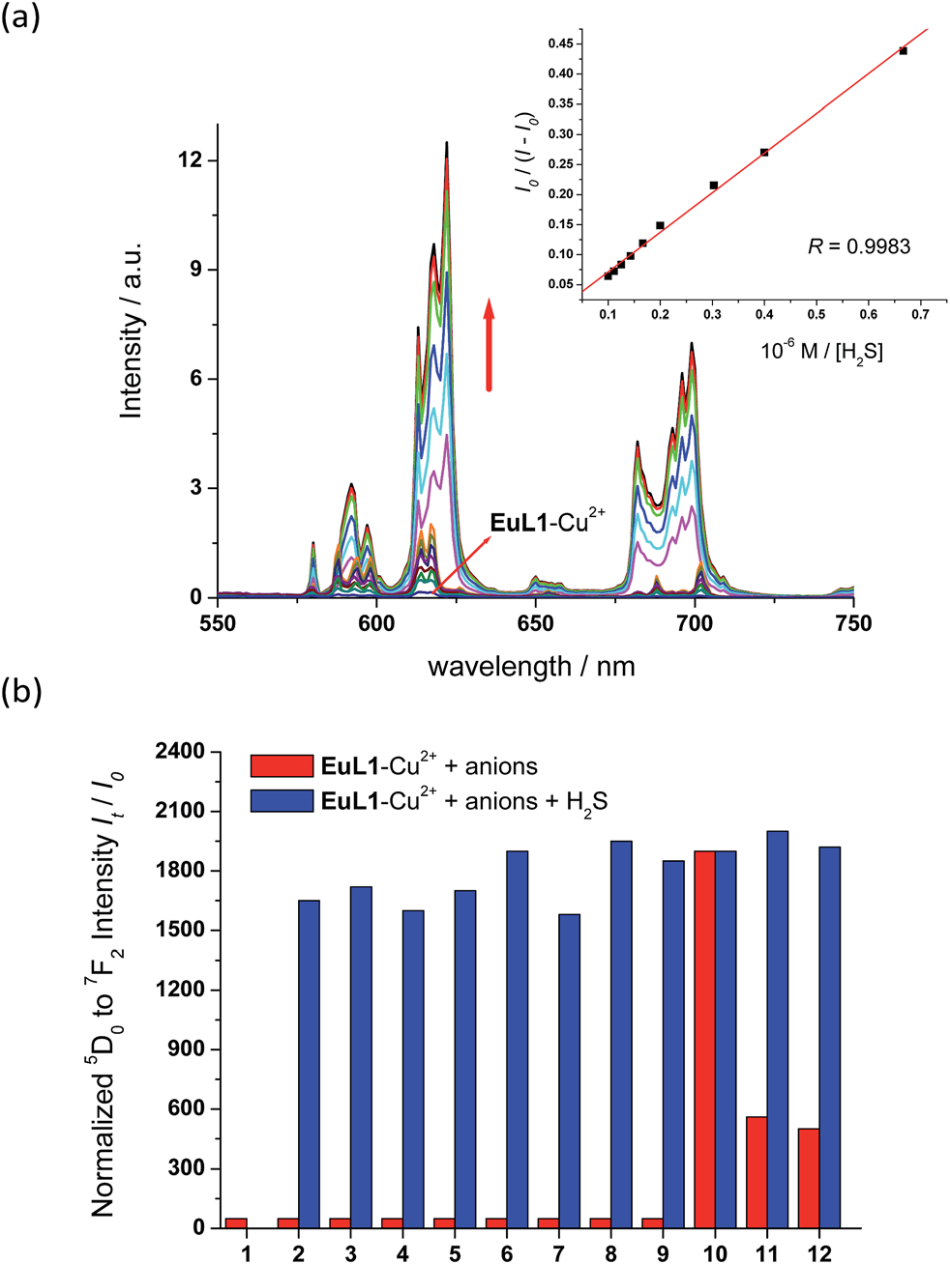
(a) Fluorimetric titration of **EuL1**–Cu^2+^ (10 μM, generated *in situ* with 2 equiv. of Cu^2+^) towards H_2_S (0–100 μM). The inset shows the plot of *I*_0_/(*I* – *I*_0_) *vs.* [Na_2_S] (0–100 μM). *I* and *I*_0_ stand for intensity of europium emission ^5^D_0_ → ^7^F_2_. (b) Effects of various anions on the luminescence intensity of **EuL1** (10 μM). 1: **EuL1** only; 2: Cl^–^; 3: SO_4_^2–^; 4: HSO_4_^–^; 5: I^–^; 6: CO_3_^2–^; 7: HPO_4_^2–^; 8: Br^–^; 9: HCO_3_^–^; 10: S^2–^; 11: GSH; 12: cysteine. All spectra were acquired in water with excitation at 325 nm.

**Fig. 6:**
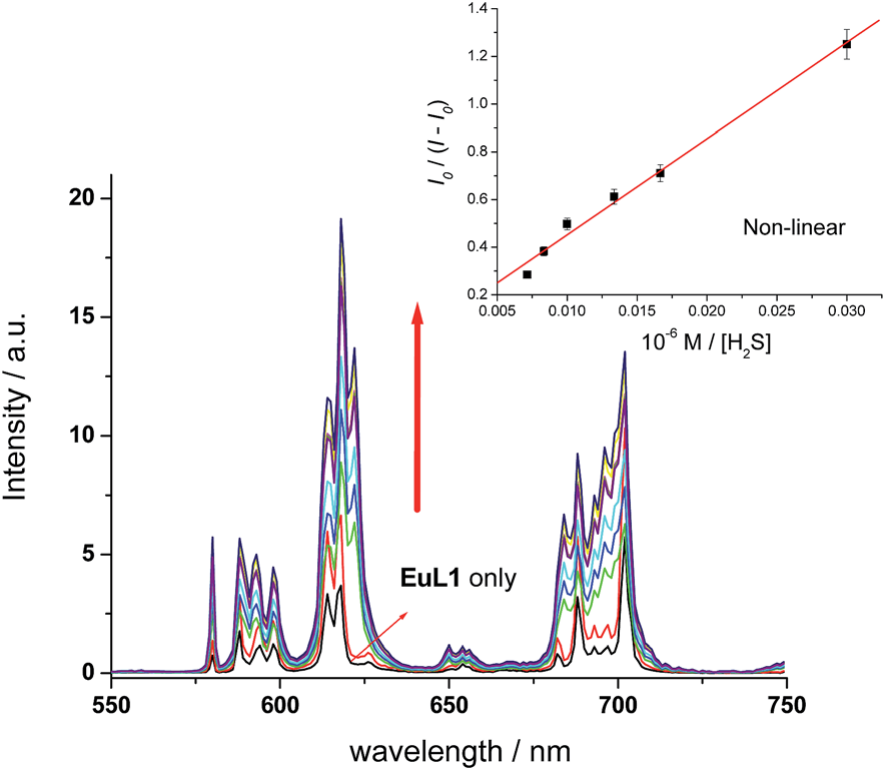
Fluorimetric titration of **EuL1** (10 μM) towards H_2_S (0–300 μM). The inset shows the plot of *I*_0_/(*I* – *I*_0_) *vs.* [H_2_S] (0–300 μM). *I* and *I*_0_ stand for intensity of europium emission ^5^D_0_ → ^7^F_2_. All spectra were acquired in water with *λ*_ex_ at 325 nm.

To study the reversibility of the binding between **EuL1** and Cu^2+^ ions, a small amount of H_2_S (Na_2_S as the source of H_2_S) was added. The **EuL1**–Cu^2+^ complex responded instantaneously (requiring only 40 s to reach saturation without stirring or shaking) (Fig. S5†), and Eu emission resumed with a similar profile for the emission spectrum to that of **EuL1** (Fig. 4). This result indicated that the DO3A–Eu^3+^ complex was not displaced by a Cu^2+^ ion, forming the **EuL1**–Cu^2+^ complex in the previous step. More interestingly, Eu emission was further enhanced (40-fold) with an excess of H_2_S and the Eu^3+^ emission profile showed significant changes, suggesting binding between **EuL1** and H_2_S (Fig. 5a). The Benesi–Hildebrand plot showed a 1 : 1 binding stoichiometry with *K*_B_ = 1.5 × 10^4^ M^–1^ (inset of Fig. 5a).^16^ The detection limit of **EuL1** towards H_2_S was calculated according to the 3*S*_D_/slope as low as 60 nM. Surprisingly, direct titration of **EuL1** against H_2_S resulted in only about a 5-fold luminescence enhancement with a non-linear relationship in the 1 : 1 Benesi–Hildebrand plot (Fig. 6). These results indicated that the Cu^2+^ ion facilitates the specific 1 : 1 binding of **EuL1** and H_2_S, presumably *via* pre-organizing the conformation of **EuL1**. On the other hand, non-specific binding (possibly a mixture of 1 : 1 and 2 : 1 binding) between **EuL1** and H_2_S resulted without the favourable conformation that is induced by the pre-complexation of a Cu^2+^ ion. This proposal was further supported by the dramatic luminescence drop of the **EuL1**–Na_2_S complex upon heating (>70 °C) (Fig. S6†). This type of Cu^2+^-assisted luminescence enhancement of Eu emission is unprecedented. In a competitive study, **EuL1**–Cu^2+^ showed insignificant changes in luminescence with a large excess of anions, including Cl^–^, SO_4_^2–^, HSO_4_^–^, I^–^, CO_3_^2–^, HPO_4_^2–^, Br^–^ and HCO_3_^–^, and only small changes for GSH and cysteine (red columns in Fig. 5b). Upon the addition of H_2_S, the Eu emissions were recovered in all the above cases, indicating a high selectivity of **EuL1**–Cu^2+^ towards H_2_S.

### Mechanistic studies

**Fig. 7:**
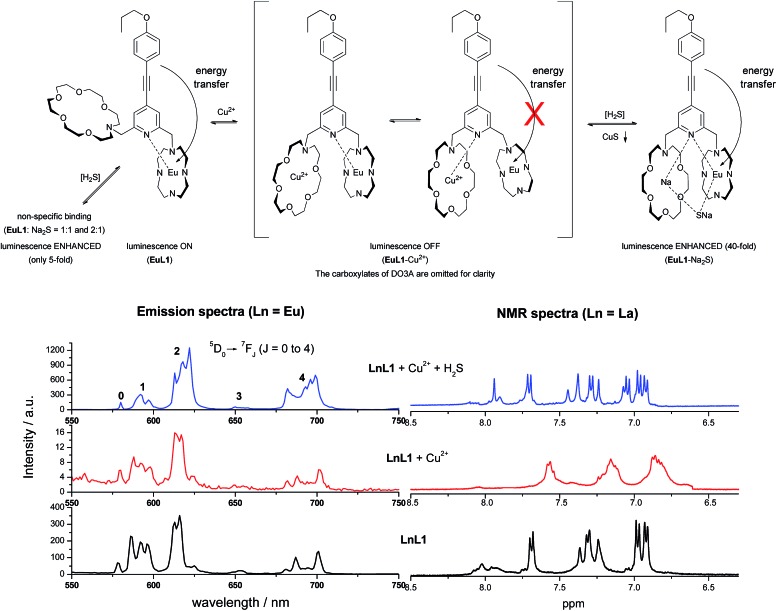
Top: proposed binding mechanism of **EuL1** towards Cu^2+^ and H_2_S (Na_2_S as the source of H_2_S). Bottom left: emission spectra of the Eu complexes (*λ*_ex_ = 325 nm). Bottom right: ^1^H NMR spectra of the La complexes (6.5–8.5 ppm).

The binding mechanisms of **EuL1** towards Cu^2+^ ions and the **EuL1**–Cu^2+^ complex towards H_2_S were studied using a comparative analysis of the emission spectra of the Eu complexes and the ^1^H NMR spectra of La complexes.^17^ As shown in Fig. 7, the profile of the emission spectrum of **EuL1** did not change significantly upon the addition of Cu^2+^ ions. Comparing [**EuL1**], [**EuL1** + Cu^2+^] and [**EuL1** + Cu^2+^ + H_2_S], measured under the same solution conditions, similar spectra were observed for [**EuL1**] and [**EuL1** + Cu^2+^] (^5^D_0_ → ^7^F_1_ : ^7^F_2_ : ^7^F_4_ of [**EuL1**] = 1 : 1.122 : 0.55 and ^5^D_0_ → ^7^F_1_ : ^7^F_2_ : ^7^F_4_ [**EuL1** + Cu^2+^] = 1 : 1.186 : 0.91, Table 1). This is correlated with the NMR data and shows that the Cu^2+^ ion is coordinated in the aza-crown. However, signal broadening was observed in the ^1^H NMR spectrum of **LaL1**, indicating rapid metal–ligand exchange. These results suggested that the pyridine moiety of the organic chromophore is rapidly switching between the DO3A–Eu^3+^ and aza-18-crown-6–Cu^2+^ complexes, causing significant luminescence quenching. Moreover, the binding of Cu^2+^ would also provide a favourable conformation for forming a new 1 : 1 complex with H_2_S. Upon the addition of H_2_S, the emission profile of **EuL1** changed significantly, Δ*J* = 2/Δ*J* = 1 for [**EuL1** + Cu^2+^ + H_2_S],^18^ and the intensity ratio was about >200% higher for [**EuL1**] and [**EuL1** + Cu^2+^]. This increase can be attributed to the lower symmetry of the complexes with the addition of sulphide ions (Fig. 7) and the ^1^H NMR signals of **LaL1** were sharpened. These results suggested new complex formation after the displacement of the Cu^2+^ ion *via* CuS precipitation. This proposal is further supported by the HRMS spectrum of the **EuL1**–Na_2_S complex (Fig. S7†) and the change in the quantum yields (Table S2†). The **EuL1**–Na_2_S complex is highly emissive probably due to its rigid structure.

**Table 1:** The ratio of ^5^D_0_ → ^7^F_*J*_ (*J* = 0 to 4) emission bands of **EuL1**, **EuL1** + Cu^2+^ and **EuL1** + Cu^2+^ + H_2_S^*a*^

^5^D_0_→	^7^F_0_	^7^F_1_	^7^F_2_	^7^F_3_	^7^F_4_
**EuL1**	0.01	1	1.22	0.08	0.55
**EuL1** + Cu^2+^	0.08	1	1.86	0.15	0.91
**EuL1** + Cu^2+^ + H_2_S	0.48	1	3.98	0.15	1.95

^*a*^All spectra were acquired in water with excitation at 325 nm.

**Fig. 8:**
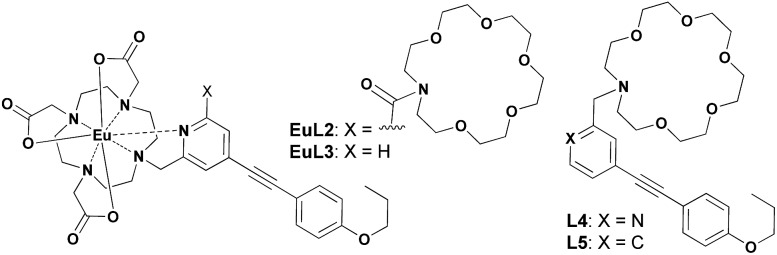
The structures of the negative control compounds **EuL2**, **EuL3**, **L4** and **L5**.

**Fig. 9:**
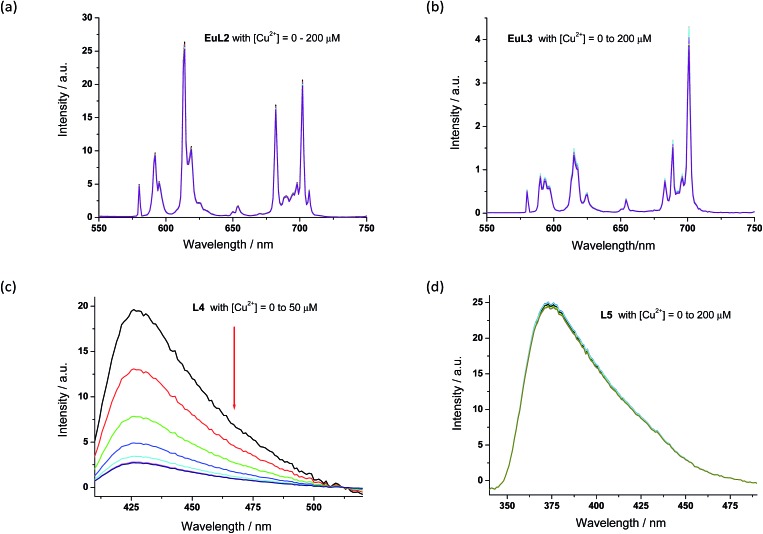
The emission spectra of negative control compounds (10 μM) with various concentration of Cu^2+^ ions. (a): **EuL2**; (b): **EuL3**; (c): **L4**; (d): **L5**. All spectra were acquired in water with *λ*_ex_ at 325 nm.

The proposed binding mechanism was also examined using a series of negative control compounds (Fig. 8).^19^**EuL2** showed no luminescence quenching upon the addition of Cu^2+^ ions (Fig. 9a). This result indicated that the carbonyl linker of aza-18-crown-6 may be too rigid for coordination between Cu^2+^ and pyridine, which could be essential for Eu emission quenching. Without the aza-crown moiety, **EuL3** also showed no luminescence quenching towards Cu^2+^ (Fig. 9b), suggesting DO3A–Eu^3+^ is stable with Cu^2+^ and the aza-crown motif is important for the Cu^2+^ binding. **L4** bearing the pyridine-chromophore showed profound luminescence quenching, but its phenyl analogue (**L5**) showed no significant change in luminescence upon the addition of Cu^2+^ ions (Fig. 9c and d). These results indicated that the pyridine moiety of the chromophore is essential for the binding of Cu^2+^ to the aza-crown moiety. The results of this series of negative control compounds are in full agreement with the proposed mechanism in Fig. 7.

## Conclusions

In summary, we have prepared a water-soluble and emissive Eu-complex (**EuL1**) based on a DO3A(Eu^3+^)–pyridine–aza-crown motif, and studied its consecutive binding properties towards Cu^2+^ and H_2_S extensively. **EuL1** binds to Cu^2+^ ions selectively (*K*_B_ = 1.2 × 10^5^ M^–1^) inducing 17-fold luminescence quenching and forming a 1 : 1 stoichiometric complex (**EuL1**–Cu^2+^), which responds to H_2_S selectively with restoration of the original **EuL1** emission followed by a further 40-fold luminescence enhancement and a nano-molar detection limit (60 nM). Mass spectroscopic analysis showed the formation of a 1 : 1 stoichiometric complex (**EuL1**–Na_2_S) with *K*_B_ = 1.5 × 10^4^ M^–1^. Without Cu^2+^ ions, **EuL1** shows non-specific binding towards H_2_S with only a 5-fold luminescence enhancement. These results indicate that the Cu^2+^ ion may pre-organize the conformation of **EuL1** and facilitate the formation of the **EuL1**–Na_2_S complex. The studies on this unprecedented Cu^2+^-assisted luminescence enhancement of Eu emission are still ongoing. With long-lived Eu emission, reversible binding properties, an instantaneous response and high selectivity towards H_2_S, this Eu-based luminescence “off–on” gate could find suitable applications for H_2_S imaging in biological systems.

## Supplementary Material

Supplementary information
