# Supplementary material for: A smart “off–on” gate for the in situ detection of hydrogen sulphide with Cu(ii)-assisted europium emission
Source: Chem Sci. 2015 Dec 7;7(3):2151–6. doi: 10.1039/c5sc04091d (PMC5968757; doi:10.1039/c5sc04091d)
Supplement: Supplementary file 1 [file SC-007-C5SC04091D-s001.pdf]

Electronic Supplementary Material (ESI) for Chemical Science.
This journal is © The Royal Society of Chemistry 2015

Supplementary Information

Table of Contents

Generation information....................S2–S3

Synthesis of **L1**, **EuL1, LaL1**, **EuL2**, **EuL3**, **L4** and **L5**....................S4–S10

Scheme S1 Synthesis of negative control compound **EuL2**....................S6

Scheme S2 Synthesis of negative control compound **EuL3**, **L4** and **L5**....................S9

NMR spectra of compounds....................S11–S23

HMRS of **L1**, **EuL1**....................S24

Table S1 and **Figure S1** HPLC analysis of **EuL1** and **EuL2**....................S25

Figure S2 UV-vis absorption spectra of **L1**, **EuL1** and excitation spectrum of **EuL1** S26

Figure S3 Job's plot of **EuL1** towards Cu^{2+} ions....................S26

Figure S4 A pH study of **EuL1** and **EuL1** with Cu^{2+} ions....................S27

Figure S5 Response time of **EuL1**-Cu^{2+} towards H_2S....................S27

Table S2 Luminescence lifetimes, number of inner sphere water molecules, quantum yield of **EuL1** alone, with Cu^{2+} ion, and with Cu^{2+} ion and H_2S....................S28

Figure S6 Temperature dependent luminescence study of the **EuL1**-Na_2S complex.......S28

Figure S7 HRMS of **EuL1** with Cu^{2+} and H_2S....................S29

Figure S8 ^{1}H NMR spectrum of **LaL1**....................S29

Figure S9 ^{1}H NMR spectrum of **LaL1**+Cu^{2+}....................S30

Figure S10 ^{1}H NMR spectrum of **LaL1**+Cu^{2+}+Na_2S....................S30

References....................S31

Generation Information

(a) Synthesis of new compounds

All air and water sensitive reactions were carried out under a nitrogen atmosphere with dry solvents under anhydrous conditions, unless otherwise noted. All the chemicals were purchased commercially and used without further purification. Anhydrous THF and toluene were distilled from sodium-benzophenone, and dichloromethane was distilled from calcium hydride. Yields refer to chromatographically, unless otherwise stated. Reactions were monitored by thin-layer chromatography (TLC) carried out on 0.25 mm silica gel plates (60F-254) that were analyzed by staining with $KMnO_4$ (200 mL H_2O of 1.5 g $KMnO_4$, 10 g K_2CO_3 and 1.25 mL of 10% aqueous NaOH), fluorescence upon 254 nm irradiation or by staining with anisaldehyde (450mL of 95% EtOH, 25 mL of conc.H_2SO_4, 15 mL of acetic acid, and 25 mL of anisaldehyde). Silica gel (60, particle size 0.040 – 0.063 mm) was used for flash chromatography. NMR spectra were recorded on either a 400 (^{1}H: 400 MHz, ^{13}C: 100 MHz), or 500 (^{1}H: 500 MHz, ^{13}C: 125 MHz). The following abbreviations were used to explain the multiplicities: s = singlet, d = doublet, t = triplet, q = quartet, m = multiplet, b = broad. High-resolution mass spectra were obtained from a MALDI-TOF Mass Spectrometer.

(b) Photophysical measurement

UV-Visible absorption spectra in the spectral range 200 to 1100 nm were recorded by an HP UV-8453 spectrophotometer. Single-photon luminescence spectra were recorded using an Edinburgh Instrument FLS920 Combined Fluorescence Lifetime and Steady state spectrophotometer that was equipped with a red sensitive single photon counting photomultiplier by Peltier Cooled Housing. The spectra were corrected for detector response and stray background light phosphorescence. The quantum yields of the compounds were measured by Demountable 142 mm (inner) diameter barium sulfide-coated integrating sphere supplied with two access ports.

(c) Binding constant and binding ratio

For the spectrofluorometric titrations, all of the solvents that were used were of analytical grade and the water that was used was purified by double distillation. Measurements were taken after equilibrium was attained and the $^5D_0 \rightarrow ^7F_2$ emission (europium) was monitored. Luminescent responses in terms of $I_o/(I - I_o)$ were plotted as a function of analyte concentration. For the determination of binding strengths of the various analyte adducts, a series of analyte solutions, at a known concentration, were mixed with the anion solutions at various concentrations. The titration curve was then fitted either with the 1:1 or 1:2 Benesi-Hildebrand equations (below) to check whether it was a 1:1 or 1:2 donor-acceptor interactions.

The 1:1 donor-acceptor interaction was analyzed by Benesi-Hildebrand equations for spectrofluorometric titration.

$$\frac{I_o}{I - I_o} = \left(\frac{a}{b-a}\right)\left(\frac{1}{K_B[substrate]} + 1\right)$$

The 1:2 donor-acceptor interaction was analyzed by Benesi-Hildebrand equations for spectrofluorometric titration.

$$\frac{I_o}{I - I_o} = \left(\frac{a}{b-a}\right)^2\left(\frac{1}{K_B[substrate]^2} + 1\right)$$

I_o and I are the luminescence intensity of the fluorogenic reagent in the absence and presence of the substrate, respectively; a and b are constants and [substrate] is the concentration of target analyte. The binding constants, K_B, were estimated from the ratio between the y-intercept to the slope that was obtained from the line of best fit by using Benesi-Hildebrand equations depending on 1:1 or 1:2 host-guest interactions respectively.[1]

(d) HPLC analysis

The reverse-phase HPLC analysis of these five europium complexes was carried out at room temperature by using an Agilent ZORBAX SB-C18 Stable Bond Analytical 4.6 X 150mm 5-micron column. The mobile phase is 0.1% formic acid in mQ water and 0.1% formic acid in acetonitrile solvent system, and the flow rate is 1.0 mL/min. Solvent gradient program is listed in the follow Table.

Time /min	0.1% CHOOH in mQ water /%	0.1% CHOOH in CH_3CN /%
0.0	95	5
14.0	60	40
15.0	60	40
20.0	0	100

Experimental Details

a) Synthesis of new compounds

*Compound **3***. To a solution of **1**[2] (1.0 g, 3.77 mmol) in THF (30 mL) and 1-ethynyl-4-propoxybenzene (**2**) (723 mg, 4.52 mmol) in Et_3N (10 mL) was added CuI (72 mg, 0.377 mmol) and $Pd(PPh_3)_2Cl_2$ (79 mg, 0.113 mmol). The dark-brown mixture was heated in the dark at 40 ℃ for 3 h. After cooling to room temperature, the reaction was quenched by the addition of a saturated NH_4Cl aqueous solution. the aqueous phase was extracted with EtOAc (3×50 mL) and then the combined organic layers were dried over $MgSO_4$, filtered, and concentrated. Silica gel flash column chromatography (hexane/ethyl acetate = 10/1 to 2/1) of the residue gave a white solid (852 mg, 78%) as the product. **3**: ^1H NMR (400 MHz, $CDCl_3$): δ 7.47 (d, *J* = 9 Hz, 2H), 7.35 (s, 2H), 6.91 (d, *J* = 9 Hz, 2H), 4.83 (s, 4H), 3.95 (t, *J* = 7 Hz, 2H), 1.88-1.80 (m, 2H), 1.05 (t, *J* = 7 Hz, 3H); ^{13}C NMR (100 MHz, $CDCl_3$): δ 160.3, 158.0, 133.7, 121.4, 114.8, 113.3, 96.6, 85.6, 69.7, 63.6, 22.8, 10.5; HRMS (ESI) m/z calcd. for $C_{18}H_{21}NO_3$ $(M+H)^+$ 298.1443, found 298.1438.

*Compound **4***. To a stirred solution of **3** (560 mg, 1.89 mmol) and Et_3N (573 mg, 5.67 mmol) in CH_2Cl_2 (25 mL) was added methanesulfonyl chloride (326 mg, 2.84 mmol) dropwise. After 20 min, the reaction was quenched by the addition of a saturated $NaHCO_3$ aqueous solution (30 mL). The organic phase was dried over $MgSO_4$, filtered, and concentrated. To a stirred solution of the residue in acetone (20 mL) at room temperature was added LiBr (12 mmol). The resulting mixture was stirred at 60 ℃ for 2 hours and then concentrated. The residue was dissolved in water (30 mL) and extracted with CH_2Cl_2 (3×30 mL). The combined organic layers were dried over $MgSO_4$, filtered, and concentrated. Silica gel flash column chromatography (hexane/ethyl acetate = 15/1 to 8/1) of the residue gave a white solid (672 mg, 84%) as the product. **4**: ^1H NMR (400 MHz, $CDCl_3$): δ 7.49 (d, *J* = 9 Hz, 2H), 7.44 (s, 2H), 6.91 (d, *J* = 9 Hz, 2H), 4.52 (s, 4H), 3.95 (t, *J* = 7 Hz, 2H), 1.88-1.79 (m, 2H), 1.054 (t, *J* = 7 Hz, 3H); ^{13}C NMR (100 MHz, $CDCl_3$): δ 160.2, 156.8, 133.6, 124.5, 114.7, 95.5, 85.1, 69.6, 33.2, 22.5, 10.5; HRMS (ESI) m/z calcd. for $C_{18}H_{17}Br_2NO$ $(M+H)^+$ 421.9755, found 421.9749.

*Compound **5***. To a stirred solution of **4** (300 mg, 0.71 mmol) and $NaHCO_3$ (270 mg, 3.55 mmol) in acetonitrile (15 mL) was added dropwise 1-aza-18-crown ether (184 mg, 0.70 mmol) at 80 ℃ for 1 hour. The resulting mixture was heated under reflux for 2 hours. After cooling to room temperature, the reaction was quenched by the addition of water. The aqueous phase was extracted with CH_2Cl_2 (3×25 mL) and the combined organic layers were dried over $MgSO_4$, filtered, and concentrated. Silica gel flash column chromatography (dichloromethane /methanol = 25/1 to 10/1) of the residue gave a white solid (175 mg, 42%) as the product. **5**: ^1H NMR (400 MHz, CD_3OD): δ 7.55 (s, 1H), 7.52 (d, *J* = 9 Hz, 2H), 7.42 (s, 1H), 6.97 (d, *J* = 9 Hz, 2H), 4.82 (s, 2H), 3.98 (t, *J* = 7 Hz, 2H), 3.79 (s, 2H), 3.73-3.53 (m, 20H), 2.76 (s, 4H), 1.88-1.80 (m, 2H), 1.05 (t, *J* = 7 Hz, 3H); ^{13}C NMR (100 MHz, CD_3OD): δ 160.4, 158.0, 134.0, 133.3, 124.5, 124.1, 114.5, 113.3, 95.1, 84.6, 69.2-66.9 (10C, crown ether), 69.3, 58.5, 54.2 (2C, crown ether), 53.4, 32.7, 22.2, 9.4; HRMS (ESI) m/z calcd. for $C_{30}H_{42}BrN_2O_6$ $(M+H)^+$ 605.2226, found 605.2221.

Compound ***L1***. To a solution of **5** (100 mg, 0.16 mmol) in acetonitrile at room temperature was added $NaHCO_3$ (61 mg, 0.80 mmol) and tri-*t*-butyl 2,2',2''-(1,4,7,10-tetraazacyclo-dodecane-1,4,7-triyl)triacetate (82 mg, 0.16 mmol). After stirring at 80 ℃ for 6 hours, the reaction was quenched with water. The aqueous phase was extracted with CH_2Cl_2 (3×25 mL) and the combined organic layers were dried over $MgSO_4$, filtered, and concentrated. Silica gel flash column chromatography (dichloromethane/methanol = 25/1 to 10/1) gave a white oil (108 mg, 60%) as the product. ^{1}H NMR (400 MHz, CD_3OD): δ 7.85 (s, 1H), 7.52 (s, 1H), 7.46 (d, *J* = 9 Hz, 2H), 6.96 (d, *J* = 9 Hz, 2H), 3.98 (t, *J* = 6 Hz, 2H), 3.88 (s, 4H), 3.73-3.53 (m, 20H), 3.50 (s, 6H), 2.83 (s, 4H), 3.20-2.30 (m, 16H), 1.88-1.80 (t, *J* = 6 Hz, 2H), 1.52-1.49 (m, 27H), 1.06 (t, *J* = 7 Hz, 3H); ^{13}C NMR (100MHz, CD_3OD): δ 173.3, 173.2, 160.3, 158.17, 133.69, 133.13, 129.00, 124.44, 123.91, 119.05, 114.80, 114.58, 114.46, 113.49, 94.60, 85.2, 82.3, 82.0, 69.4-67.5 (10C, crown ether), 69.3, 59.9, 59.3, 55.9-55.5 (8C, DO3A), 54.2 (2C, crown ether), 53.5, 27.1, 27.1, 22.2, 9.4; HRMS (ESI) m/z calcd. for $C_{56}H_{91}N_6O_{12}$ $(M+H)^+$ 1039.6695, found 1039.6689.

Compound ***EuL1.*** **L1** (30 mg, 0.029 mmol) was dissolved in TFA (2 mL) and the mixture was stirred at room temperature under argon for 12 hours. After removal of the volatiles, the light-yellow residue was dissolved in CH_2Cl_2 and concentrated to ensure the removal of TFA. Hydrolysis of the *t*-butyl ester groups was confirmed by ^{1}H NMR of the crude product. To a stirred solution of the residue in methanol (2 mL) was added $EuCl_3{\bullet}6H_2O$ (0.032 mmol) followed by adjustment of the pH to 5.8. The mixture was stirred at room temperature for 30 h and then concentrated. Recrystallization of the residue from CH_3CN gave an off-white solid (17.4 mg, 60%) as the product. **EuL1**: HRMS (ESI) m/z calcd. for $C_{44}H_{63}EuN_6NaO_{12}$ $(M+Na)^+$ 1043.3614, found 1043.3680.

Compound ***LaL1.*** **L1** (30 mg, 0.029 mmol) was dissolved in TFA (2 mL) and the mixture was stirred at room temperature under argon for 12 hours. After removal of the volatiles, the light-yellow residue was dissolved in CH_2Cl_2 and concentrated to ensure the removal of TFA. Hydrolysis of the *t*-butyl ester groups was confirmed by ^{1}H NMR of the crude product. To a stirred solution of the residue in methanol (2 mL) was added $La(OTf)_3$ (0.030 mmol) followed by adjustment of the pH to 5.8. The mixture was stirred at room temperature for 30 h and then concentrated. Recrystallization of the residue from CH_3CN gave an off-white solid (18.7 mg, 65%) as the product. **LaL1**: HRMS (ESI) m/z calcd. for $C_{44}H_{63}LaN_6NaO_{12}$ $(M+Na)^+$ 1029.3465, found 1029.3413.

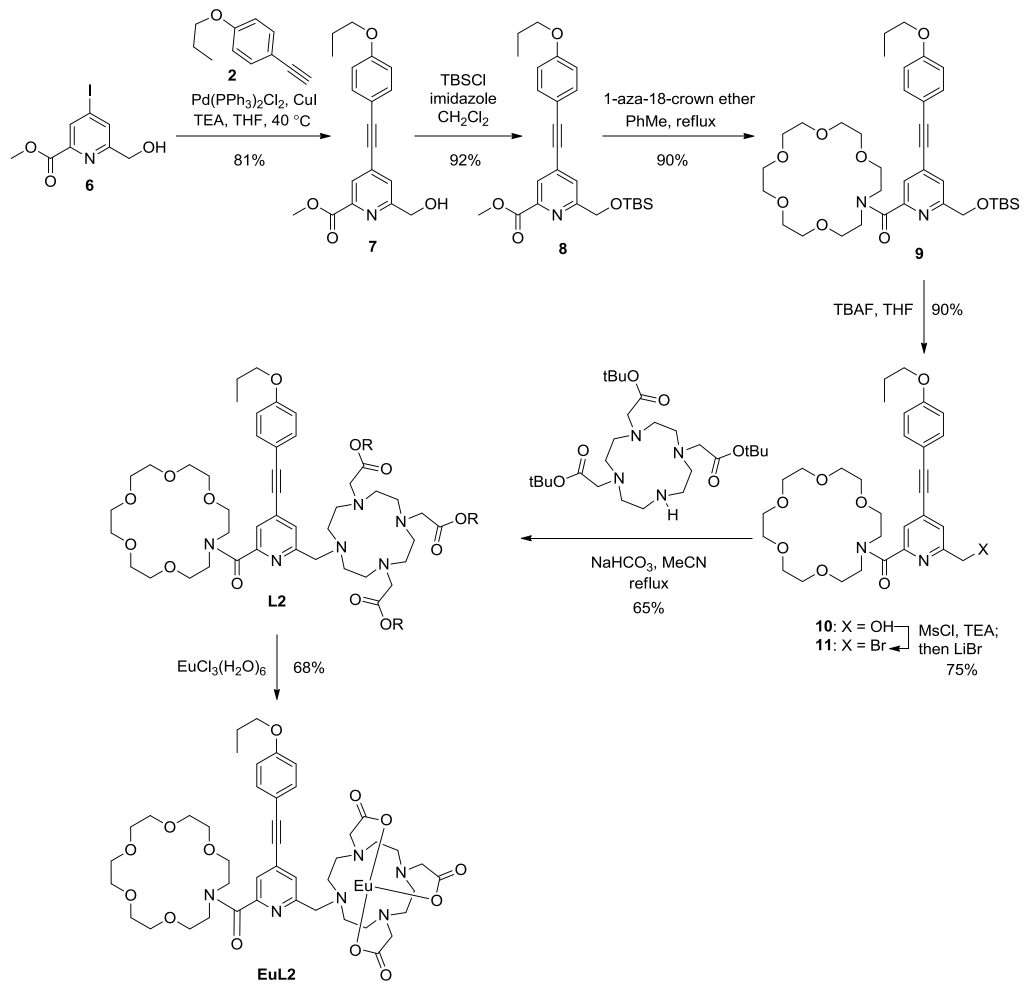

Scheme S1 The synthesis of negative control compound **EuL2**

*Compound **7***. To a solution of **6**[3] (2.0 g, 6.82 mmol) in THF (30 mL) at room temperature was added a solution of 1-ethynyl-4-propoxybenzene (**2**) (1.20 g, 7.50 mmol) in Et_3N (10 mL) and then CuI (0.130 g, 0.682 mmol) and $Pd(PPh_3)_2Cl_2$ (0.143 g, 0.205 mmol). The resulting dark-brown mixture was heated in the dark at 40 ℃ for 4 hours. After cooling to room temperature, the reaction was quenched by the addition of a saturated NH_4Cl aqueous solution. The aqueous phase was extracted with EtOAc (3×50 mL) and the combined organic layers were dried over $MgSO_4$, filtered, and concentrated. Silica gel flash column chromatography (hexane/ethyl acetate = 10/1 to 2/1) of the residue gave a white solid (1.80 g, 81%) as the product. **7**: ^{1}H NMR (400 MHz, $CDCl_3$): δ 8.08 (s, 1H), 7.58 (s, 1H), 7.48 (d, *J* = 9 Hz, 2H), 6.90 (d, *J* = 9 Hz, 2H), 4.86 (s, 2H), 4.01 (s, 3H), 3.95 (t, *J* = 7 Hz, 2H), 1.85-1.78 (m, 2H), 1.05 (t, *J* = 7 Hz, 3H); ^{13}C NMR (100 MHz, $CDCl_3$): δ 165.3, 160.3, 147.2, 134.0, 133.7, 125.7, 125.3, 114.8, 113.5, 96.1, 85.1, 69.7, 64.6, 53.0, 22.5, 10.5; HRMS (ESI) m/z calcd. for $C_{19}H_{20}NO_4$ $(M+H)^+$ 326.1392, found 326.1382.

*Compound **8***. To a stirred solution of **7** (1.0 g, 3.10 mmol) in CH_2Cl_2 (15 mL) at room temperature was added imidazole (253 mg, 3.73 mmol) and TBSCl (560 mg, 3.73 mmol). After stirring at room temperature for 2 hours, the reaction was quenched by addition of

water (20 mL). The aqueous phase was extracted with CH_2Cl_2 (3×20 mL) and the combined organic layers were dried over $MgSO_4$, filtered, and concentrated. Silica gel flash column chromatography (hexane/ethyl acetate = 15/1 to 10/1) of the residue afforded a pale yellow oil (1.25 g, 92%) as the product. **8**: ^{1}H NMR (400 MHz, $CDCl_3$): δ 8.06 (s, 1H), 7.75 (s, 1H), 7.51 (d, *J* = 9 Hz, 2H), 6.90 (d, *J* = 9 Hz, 2H), 4.93 (s, 2H), 4.01 (s, 3H), 3.96 (t, *J* = 7 Hz, 2H), 1.84-1.80 (m, 2H), 1.05 (t, *J* = 7 Hz, 3H), 0.98 (s, 9H), 0.15 (s, 6H); ^{13}C NMR (400MHz, $CDCl_3$): δ 165.5, 162.3, 160.2, 147.1, 133.8, 133.7, 125.3, 124.7, 113.7, 95.4, 85.6, 69.7, 65.9, 53.1, 30.6, 26.0, 25.7, 22.5, 10.5, -3.6; HRMS (ESI) m/z calcd. for $C_{25}H_{34}NO_4Si$ $(M+H)^+$ 440.2257, found 440.2284.

Compound ***9***. To a stirred solution of **8** (500 mg, 1.14 mmol) in toluene (10 mL) at room temperature was added 1-aza-18-crown ether (358 mg, 1.37 mmol) and Et_3N (23 mg, 0.23 mmol). The resulting mixture was heated under reflux for 6 hours and then concentrated. Silica gel flash column chromatography (dichloromethane/methanol = 40/1 to 20/1) of the residue gave a pale yellow oil (688 mg, 90%) as the product. **9**: ^{1}H NMR (400 MHz, $CDCl_3$): δ 7.57 (s, 1H), 7.53 (s, 1H), 7.48 (d, *J* = 9 Hz Hz, 2H), 6.89 (d, *J* = 9 Hz, 2H), 4.82 (s, 2H), 3.95 (t, *J* = 7 Hz, 2H), 3.82 (m, 4H), 3.75-3.55 (m, 20H), 1.82 (m, 2H), 1.067, 1.04 (t, *J* = 7 Hz, 3H), 0.977 (s, 9H), 0.145 (s, 6H). ^{13}C NMR (100 MHz, $CDCl_3$): δ 168.7, 160.3, 160.1, 133.9, 133.6, 129.5, 123.5, 122.5, 115.4, 114.7, 113.7, 95.3, 85.8, 70.7-69.5 (10 C, crown ether), 69.3, 65.6, 52.16, 49.8, 47.2, 29.7, 29.4, 26.0, 25.1, 22.5, 10.5, -5.3; HRMS (ESI) m/z calcd. for $C_{36}H_{55}N_2O_8Si$ $(M+H)^+$ 671.3728, found 671.3785.

Compound ***10***. To a stirred solution of **9** (688 mg, 1.03 mmol) in THF (10 mL) at room temperature was added TBAF (2 ml, 1.0 M THF solution). The resulting mixture was stirred at room temperature for 1 hour and then concentrated. Silica gel flash column chromatography (dichloromethane/methanol = 30/1 to 15/1) of the residue gave a white solid (515 mg, 90%) as the product. **10**: ^{1}H NMR (400 MHz, $CDCl_3$): δ 7.53 (s, 1H), 7.41 (d, *J* = 9 Hz, 2H), 7.35 (s, 1H), 6.83 (d, *J* = 9 Hz, 2H), 4.67 (s, 2H), 4.07 (s, 1H), 3.89 (t, *J* = 7 Hz, 2H), 3.78-3.54 (m, 24H), 1.80-1.73 (m, 2H), 1.00 (t, *J* = 7 Hz, 3H); ^{13}CNMR (100 MHz, $CDCl_3$): δ 168.8, 160.1, 159.2, 153.6, 133.6, 123.8, 122.5, 114.7, 113.7, 95.4, 85.5, 70.7-69.5 (10 C, crown ether), 69.3, 64.4, 49.9, 47.2, 22.5, 10.5; HRMS (ESI) m/z calcd. for $C_{18}H_{20}NO_3$ $(M+H)^+$ 557.2863, found 557.2862.

Compound ***11***. To a stirred solution of **10** (500 mg, 0.90 mmol) and Et_3N (273 mg, 2.70 mmol) in CH_2Cl_2 (25 mL) at room temperature was added methanesulfonyl chloride (155 mg, 1.35 mmol) dropwise. After stirring at room temperature for 20 min, the reaction was quenched by the addition of a saturated $NaHCO_3$ aqueous solution (30 mL). The organic phase was separated, dried over $MgSO_4$, filtered and concentrated. To a stirred solution of the crude product in acetone (20 mL) at room temperature was added LiBr (1.8 mmol). The resulting mixture was stirred at 60 ℃ for 2 hours and then concentrated. The residue was dissolved in water (30 mL) and extracted with CH_2Cl_2 (3×30 mL). The combined organic layers were dried over $MgSO_4$, filtered, and concentrated. Silica gel flash column chromatography (dichloromethane/methanol = 30/1 to 15/1) of the residue gave a white solid (415 mg, 75%) as the product. **11**: ^{1}H NMR (400 MHz, CD_3OD): δ 7.63 (s, 1H), 7.60 (s, 1H), 7.52 (d,

J = 9 Hz, 2H), 6.96 (d, J = 9 Hz, 2H), 4.72, 4.61 (s, 2H), 3.98 (t, J = 7 Hz, 2H), 3.88-3.55 (m, 24H), 1.88-1.78 (m, 2H), 1.05 (t, J = 7 Hz, 3H); ^{13}C NMR (100 MHz, CD_3OD): δ 169.2, 160.5, 156.7, 154.4, 134.2, 133.4, 125.2, 124.8, 123.9, 123.8, 114.5, 113.2, 95.7, 84.3, 70.2-68.7 (10 C, crown ether), 69.3, 49.7, 47.15, 32.0, 22.2, 9.4; HRMS (ESI) m/z calcd. for $C_{30}H_{40}BrN_2O_7$ $(M+H)^+$ 619.2019, found 619.2017.

*Compound **L2.*** To a solution of **11** (150 mg, 0.24 mmol) in acetonitrile (30 mL) at room temperature was added $NaHCO_3$ (92 mg, 1.21 mmol) and DO3A(O^{t}Bu)$_3$ (123 mg, 0.24 mmol). After stirring at 80 ℃ for 6 hours, the reaction was quenched by addition of water (30 mL). The aqueous phase was extracted with CH_2Cl_2 (3×25 mL). The combined organic layers were dried over $MgSO_4$, filtered, and concentrated. Silica gel flash column chromatography (dichloromethane/methanol = 25/1 to 10/1) of the residue gave a white oil (164 mg, 65%) as the product. **12**: ^{1}H NMR (400 MHz, $CDCl_3$): δ 7.91 (s, 1H), 7.57 (s, 1H), 7.36 (d, J = 9 Hz, 2H), 6.84 (d, J = 9 Hz, 2H), 3.95 (t, J = 7 Hz, 2H), 3.88 (s, 2H), 3.81-3.61 (m, 24H), 3.12 (s, 6H), 3.05-2.40 (m, 16H, *cyclen*), 1.88-1.80 (m, 2H), 1.44 (s, 27H), 1.06 (t, J = 7 Hz, 3H); ^{13}C NMR (100 MHz, $CDCl_3$): δ 173.5, 172.7, 168.6, 160.1, 157.0, 154.1, 133.9, 133.6, 133.4, 126.1, 123.9, 114.6, 113.6, 95.3, 85.3, 82.8, 82.3, 70.3 (10 C, crown ether), 69.7, 60.2, 56.2, 55.8, 53.5, 51.1, 50.2, 49.86, 46.8, 28.0, 27.8, 22.5, 10.5; HRMS (ESI) m/z calcd. for $C_{56}H_{89}N_6O_{13}$ $(M+H)^+$ 1053.6488, found 1053.6480.

*Compound **EuL2***. **L2** (30 mg, 0.028 mmol) was dissolved in TFA (2 mL) and the resulting mixture was stirred at room temperature under argon for 12 hours. After removal of TFA, the pale yellow residue was dissolved in CH_2Cl_2 and concentrated to ensure the removal of TFA. Hydrolysis of the *t*-butyl ester groups was confirmed by ^{1}H NMR of the crude product. The residue was then dissolved in water/methanol (2:1 v/v, 3 mL) and treated with $EuCl_3•6H_2O$ (0.028 mmol). After adjustment of the pH to 7.0 with sodium hydroxide, the mixture was stirred at room temperature for 30 hours and then concentrated. Recrystallization of the residue from CH_3CN gave a pale white solid (20 mg, 68%) as the product. **EuL2**: HRMS (ESI) m/z calcd. for $C_{44}H_{61}EuN_6NaO_{13}$ $(M+Na)^+$ 1057.3407, found 1057.3483.

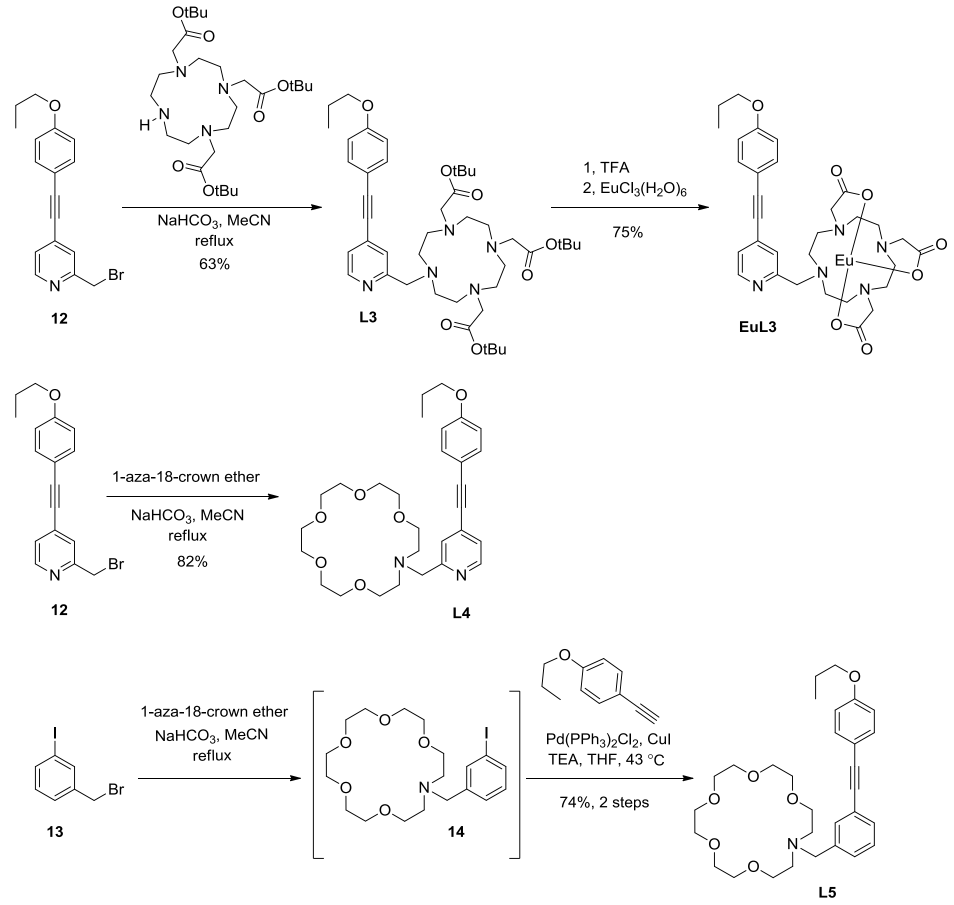

Scheme S2 The synthesis of negative control compounds **EuL3**, **L4** and **L5**

*Compound **L3***. To a solution of **12**[4] (80 mg, 0.24 mmol) in acetonitrile (15 mL) at room temperature, was added K_2CO_3 (92 mg, 1.21 mmol) and DO3A(O*t*Bu)$_3$ (123 mg, 0.24 mmol). After stirring at 80 ℃ for 6 hours, the reaction was quenched by addition of water (15 mL). The aqueous phase was extracted with CH_2Cl_2 (3×15 mL). The combined organic layers were dried over $MgSO_4$, filtered, and concentrated. Silica gel flash column chromatography (dichloromethane/methanol = 30/1 to 20/1) of the residue gave an off-white oil (124 mg, 68%) as the product. ^{1}H NMR (500 MHz, $CDCl_3$) δ 8.20 (d, J = 7 Hz, 1H), 7.44 (d, J = 7 Hz, 2H), 7.24 (s, 1H), 7.17 (d, J = 7 Hz, 1H), 6.86 (d, J = 7 Hz, 2H), 3.93 (t, J = 7 Hz, 2H), 3.57 (s, 2H), 3.21-2.30 (m, 22H), 1.80-1.76 (m, 2H), 1.46 (s, 18H), 1.38 (s, 9H), 1.05 (t, J = 7 Hz, 3H); ^{13}C NMR (125 MHz, $CDCl_3$) δ 172.7, 172.4, 160.1, 158.8, 148.6, 133.4, 132.8, 124.9, 123.6, 114.7, 113.5, 95.1, 85.2, 81.9, 81.8, 69.6, 58.7, 56.3, 55.5, 53.3, 50.3, 27.9, 22.4, 10.3; HRMS (ESI) m/z calcd. for $C_{43}H_{66}N_5O_7$ $(M+H)^+$ 764.4962, found 764.4961.

*Compound **EuL3***. **L3** (0.020 mmol) was dissolved in TFA (1 mL) and the mixture was stirred at room temperature under argon for 12 hours. After removal of TFA under reduced pressure, the pale yellow residue was dissolved in CH_2Cl_2, and concentrated to ensure the removal of TFA. Hydrolysis of the *t*-butyl ester groups was confirmed by ^{1}H NMR of the crude product. The residue was then dissolved in water/methanol (2:1 v/v, 3 mL) and treated with $EuCl_3·(H_2O)_6$ (0.020 mmol). After adjustment of the pH to 7.0 with a 1 N aqueous sodium hydroxide solution, the mixture was stirred at 70 ℃ for 24 hours and then concentrated.

Recrystallization of the residue from CH_3CN gave an off-white solid (75%) as the product. **EuL3**: HRMS (ESI) m/z calcd. for $C_{31}H_{41}EuN_5O_8$ $(M+H_2O+H)^+$764.2167, found 764.2221, $(M+H)^+$746.2062, found 746.2051;

*Compound **L4***. To a stirred solution of **12**[4] (235 mg, 0.71 mmol) and $NaHCO_3$ (270 mg, 3.55 mmol) in acetonitrile (15 mL) was added 1-aza-18-crown ether (184 mg, 0.71 mmol) at 80 ℃ for 1 hour. The resulting mixture was heated under reflux for 2 hours, and the reaction was quenched by the addition of water. The aqueous solution was extracted with CH_2Cl_2 (3×25 mL) and then the combined organic layer were dried over $MgSO_4$, filtered, and concentrated. Silica gel flash column chromatography (dichloromethane/methanol = 40/1 to 30/1) gave a white solid (299 mg, 82%). **L4**: ^{1}H NMR (400 MHz, $CDCl_3$): δ 8.54 (d, *J* = 5 Hz, 1H), 7.46 (d, *J* = 9 Hz, 2H), 7.28 (d, *J* = 4 Hz, 2H), 6.88 (d, *J* = 9 Hz, 2H), 3.92 (t, *J* = 7 Hz, 2H), 3.78-3.59 (m, 22H), 1.84-1.75 (m, 2H), 1.01 (t, *J* = 7 Hz, 3H); ^{13}C NMR (100 MHz, $CDCl_3$): δ 160.2, 149.9, 133.6, 133.3, 125.6, 124.5, 114.7, 113.5, 85.3, 69.6, 69.2, 69.0, 69.0, 68.9, 68.8, 68.8, 68.8, 66.7, 59.1, 53.8, 29.7, 22.5, 10.5, 1.0; HRMS (ESI) m/z calcd. for $C_{29}H_{40}N_2O_6$ $(M+H)^+$ 513.2966, found 513.2956.

*Compound **L5***. To a stirred solution of **13** (133 mg, 0.45 mmol) in acetonitrile (5 mL) was added $NaHCO_3$ (76 mg, 95 mmol) and 1-aza-18-crown-6 ether (120 mg, 0.45 mmol). The resulting mixture was stirred under refluxing condition for 12 hour. The mixture was then cooled to room temperature and washed with water (5 mL). The aqueous phase was extracted with EtOAc (3×25 mL) and the combined organic layers were dried over MgSO4, filtered, and concentrated. To a solution of crude **14** in THF (20 mL) and 1-ethynyl-4-propoxybenzene (72.3 mg, 0.45 mmol) in Et_3N (1 mL) was added CuI (7.2 mg, 0.038 mmol) and $Pd(PPh_3)_2Cl_2$ (7.9 mg, 0.01 mmol). The dark-brown mixture was heated in the dark at 40 ℃ for 3 hour. After cooling to room temperature, the reaction was quenched by the addition of saturated NH_4Cl aqueous solution. the aqueous phase was extracted with EtOAc (3×25 mL) and then the combined organic layers were dried over $MgSO_4$, filtered, and concentrated. Silica gel flash column chromatography (dichloromethane/methanol = 40/1 to 30/1) of the residue gave a white solid (170 mg, 74% from **13**) as the product. **L5**: ^{1}H NMR (400 MHz, $CDCl_3$): δ 7.52 (s, 1H), 7.46-7.27 (m, 5H), 6.88 (d, *J* = 9 Hz, 2H), 3.94 (t, *J* = 7 Hz, 4H), 3.69-3.62 (m, 20H), 2.94 (s, 4H), 1.86-1.78 (m, 2H), 1.04 (t, *J* = 7 Hz, 3H); ^{13}C NMR (75 MHz, $CDCl_3$): δ 159.3, 133.0, 132.4, 130.6, 129.4, 128.5, 123.7, 115.0, 114.5, 87.9, 70.5, 70.1, 69.6, 53.8, 29.70, 22.5, 10.5; HRMS (ESI) m/z calcd. for $C_{30}H_{41}NO_6$ $(M+H)^+$ 512.3014, found 512.3006.

^{1}H NMR and ^{13}C NMR spectra of **3**

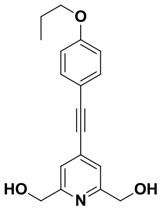

3

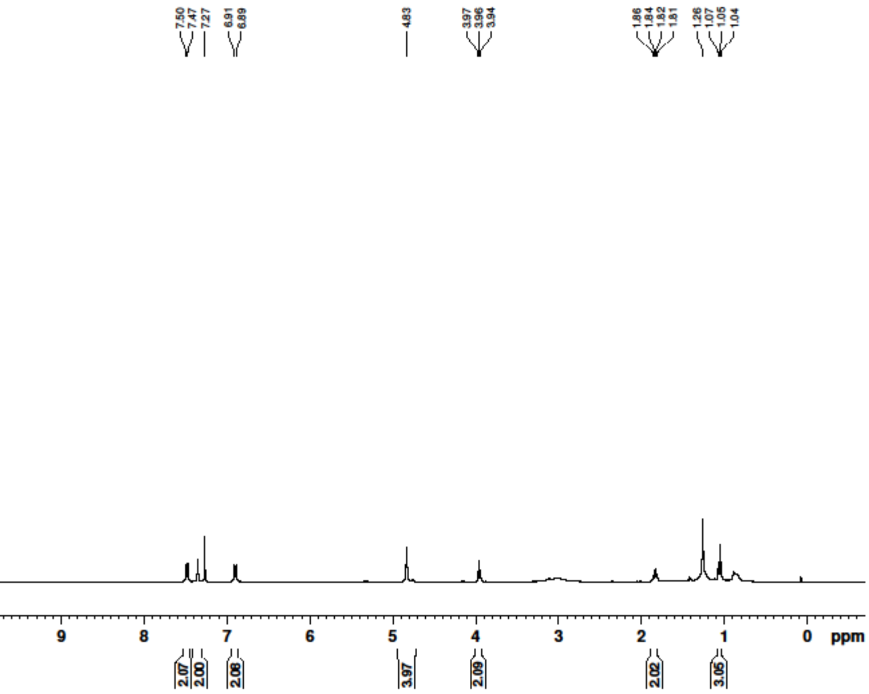

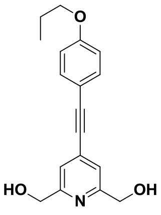

3

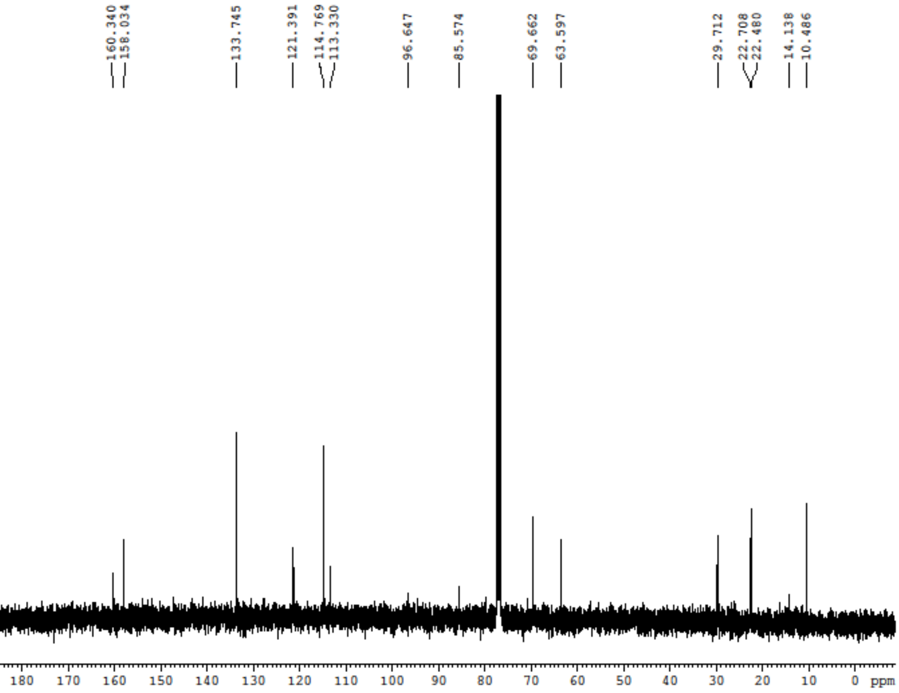

^{1}H NMR and ^{13}C NMR of spectra of **4**

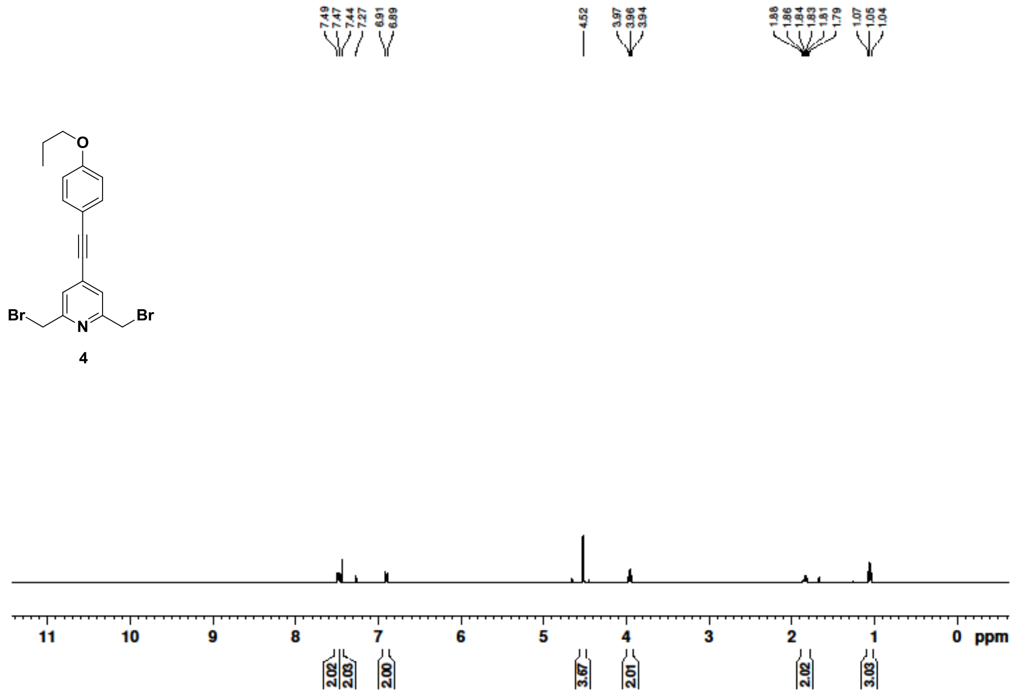

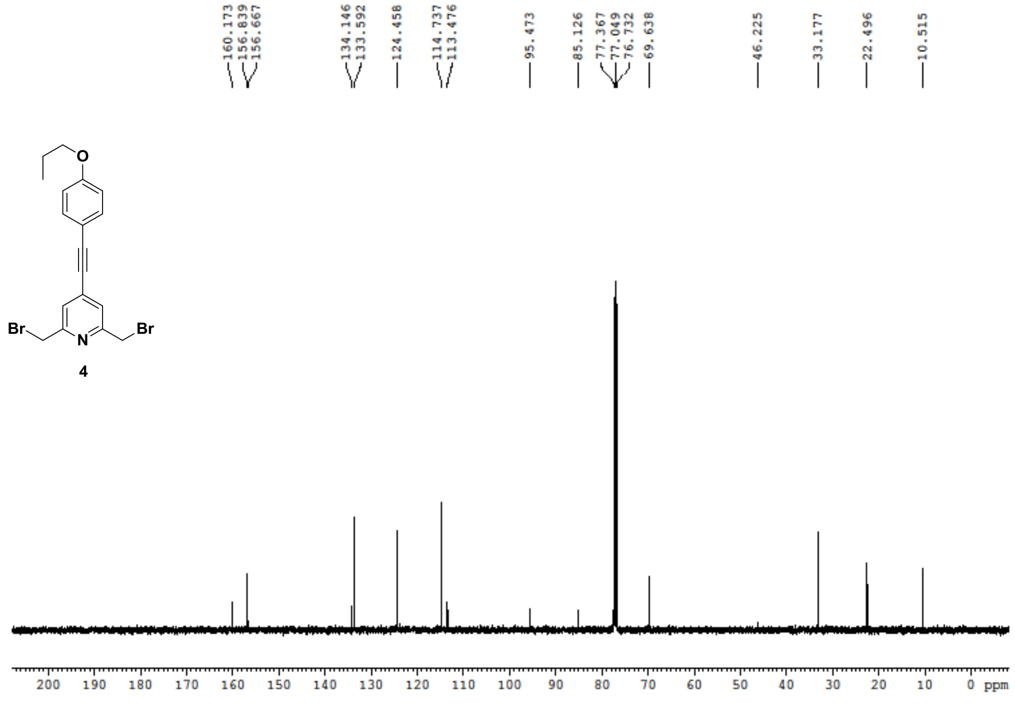

^{1}H NMR and ^{13}C NMR spectra of **5**

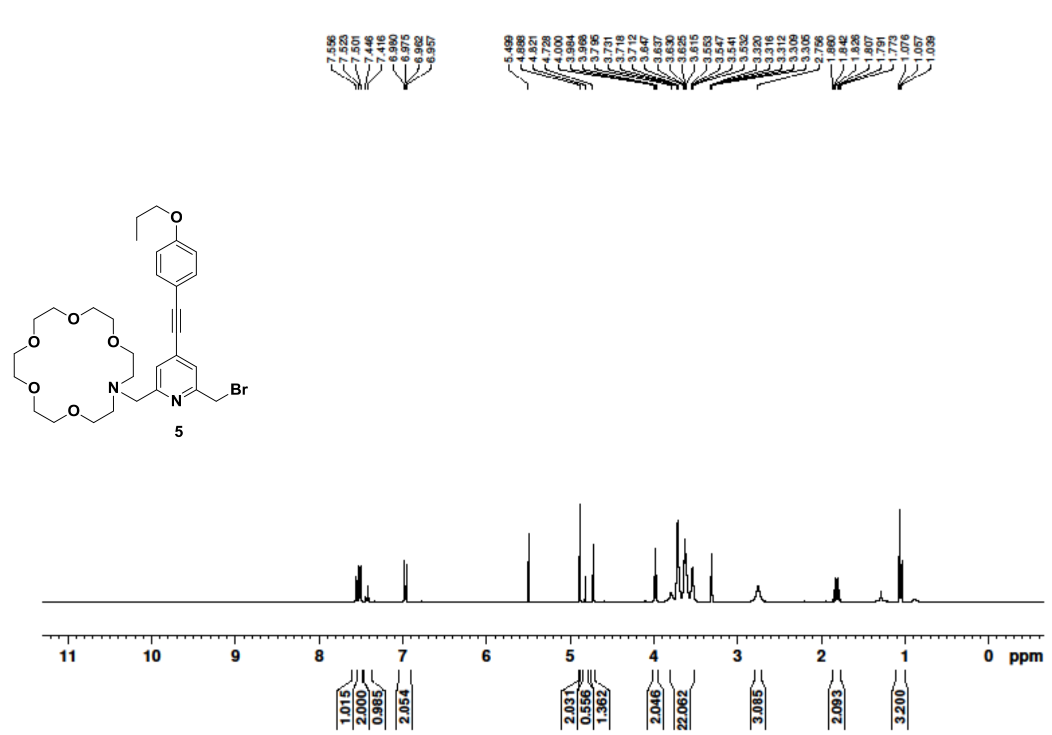

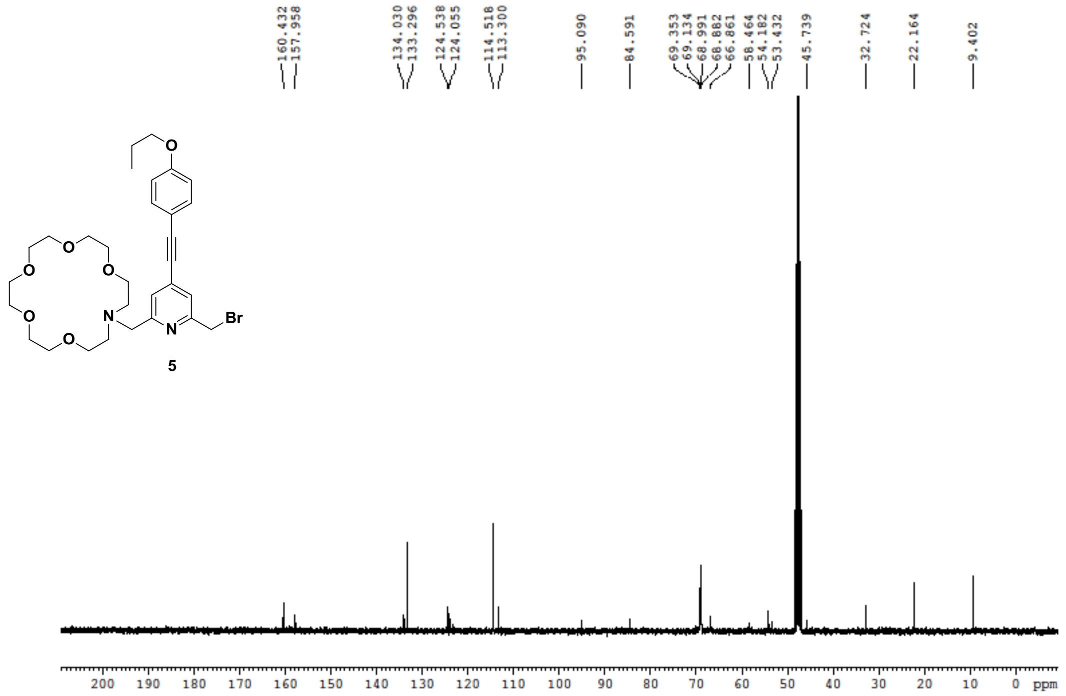

^{1}H NMR and ^{13}C NMR spectra of **L1**

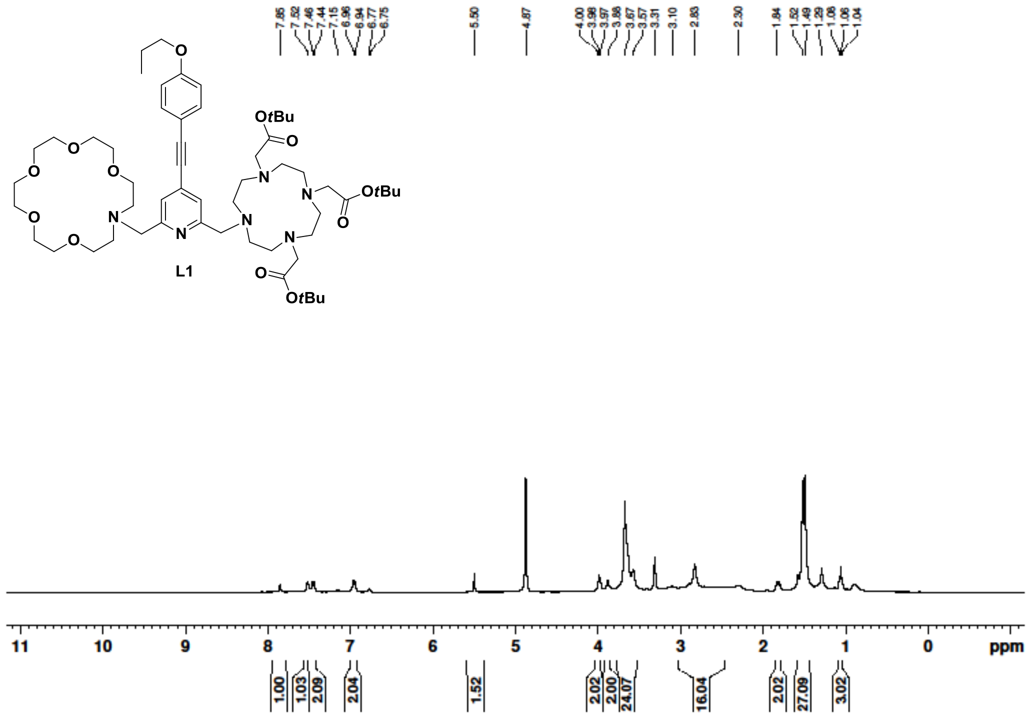

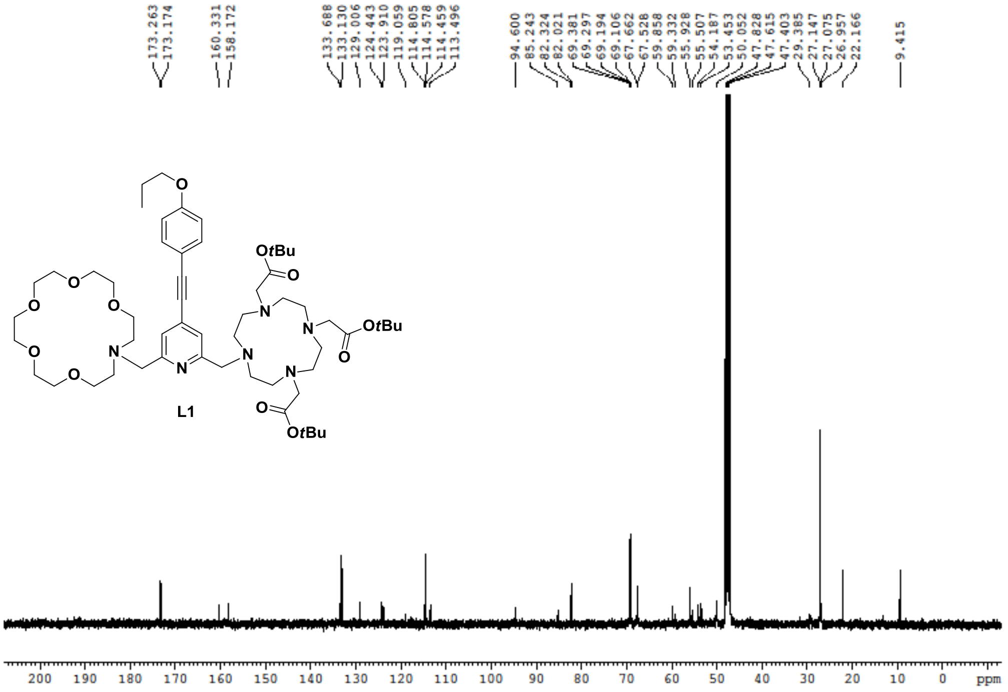

^{1}H NMR and ^{13}C NMR spectra of **7**

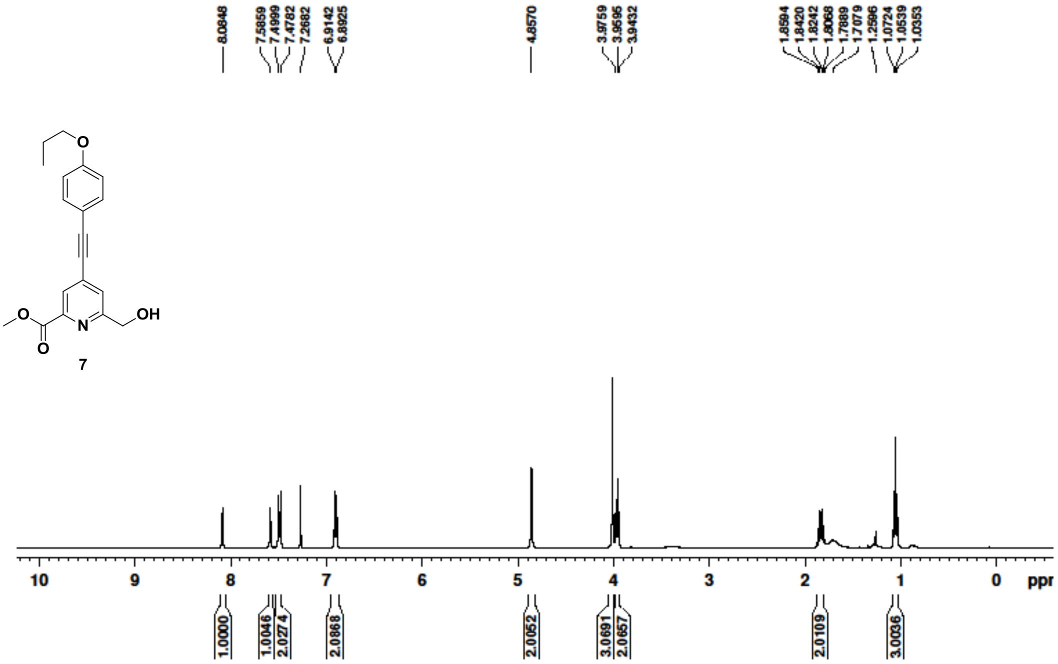

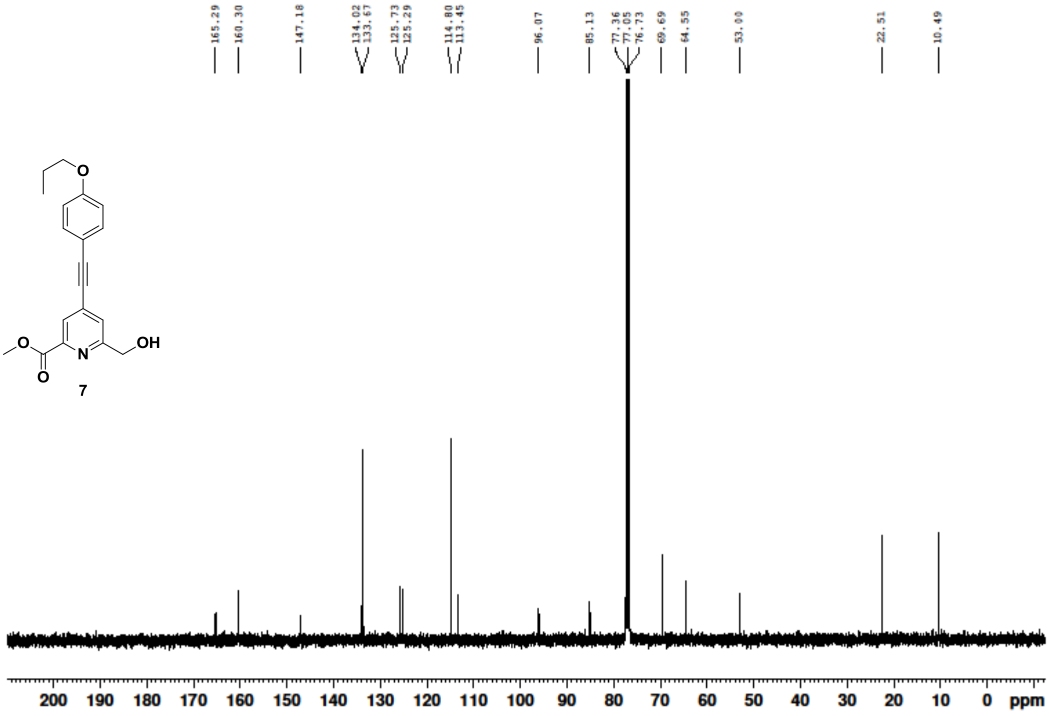

^{1}H NMR and ^{13}C NMR spectra of **8**

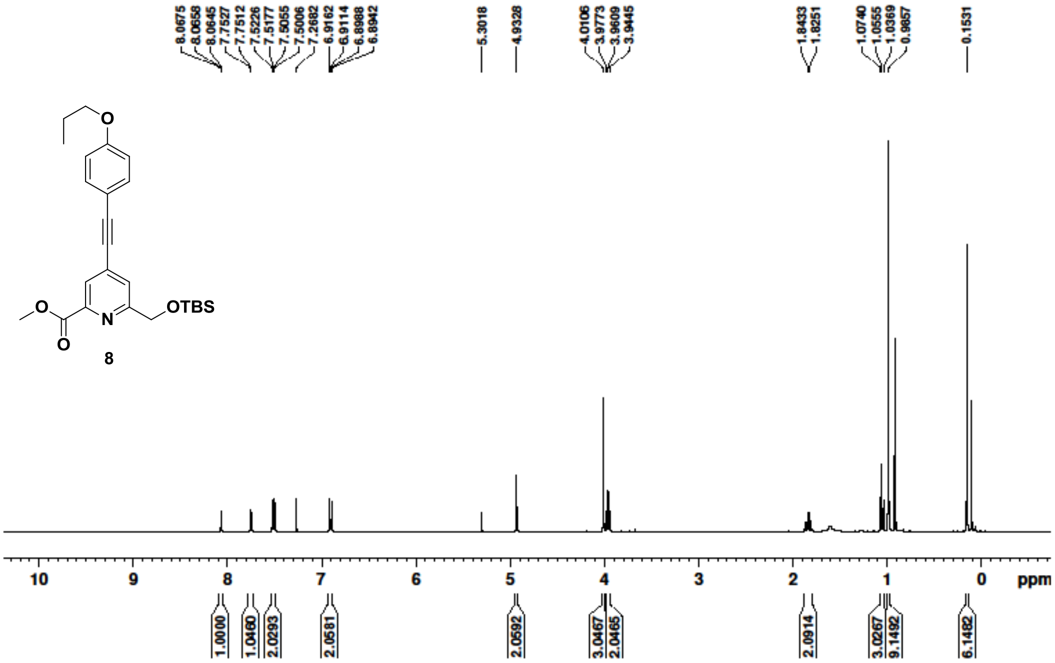

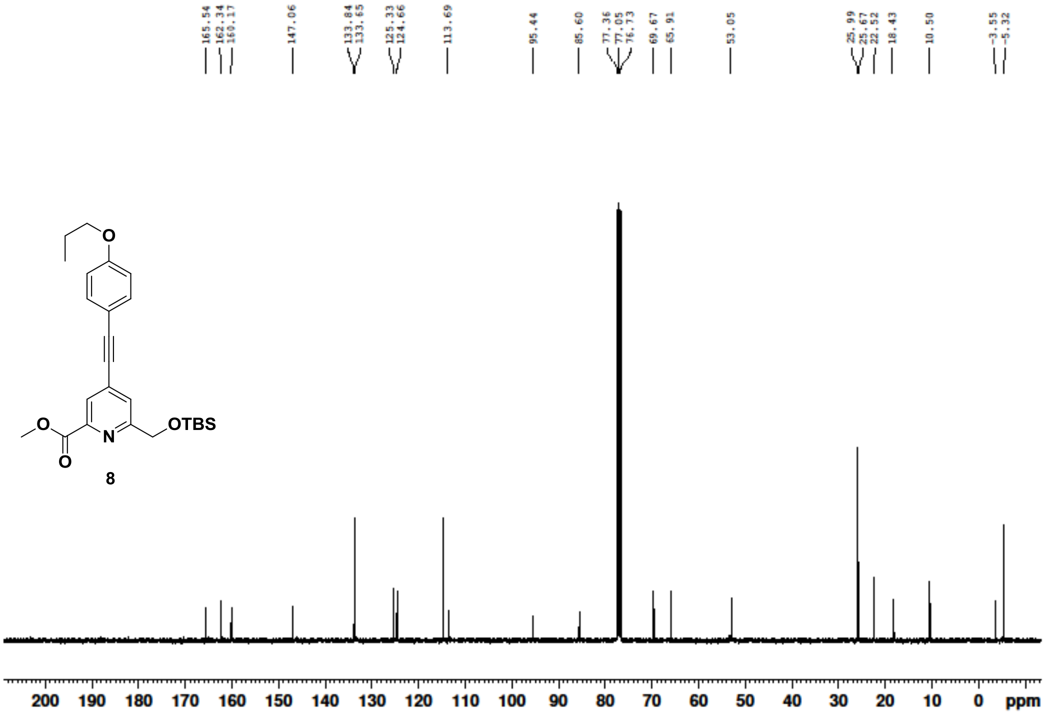

^{1}H NMR and ^{13}C NMR spectra of **9**

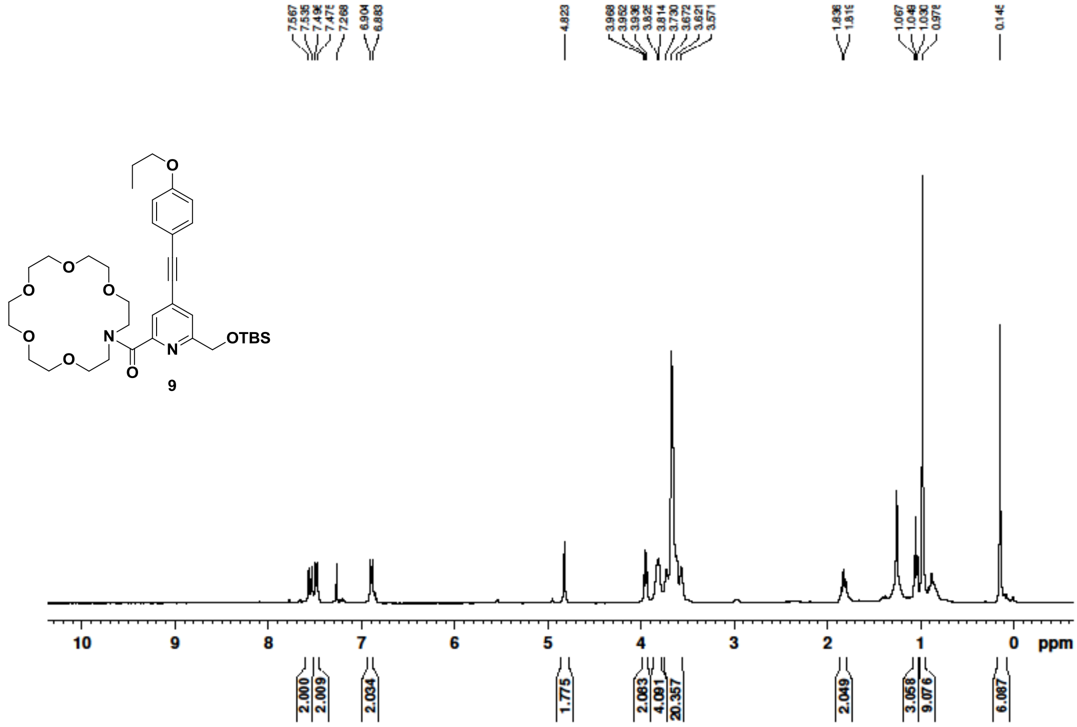

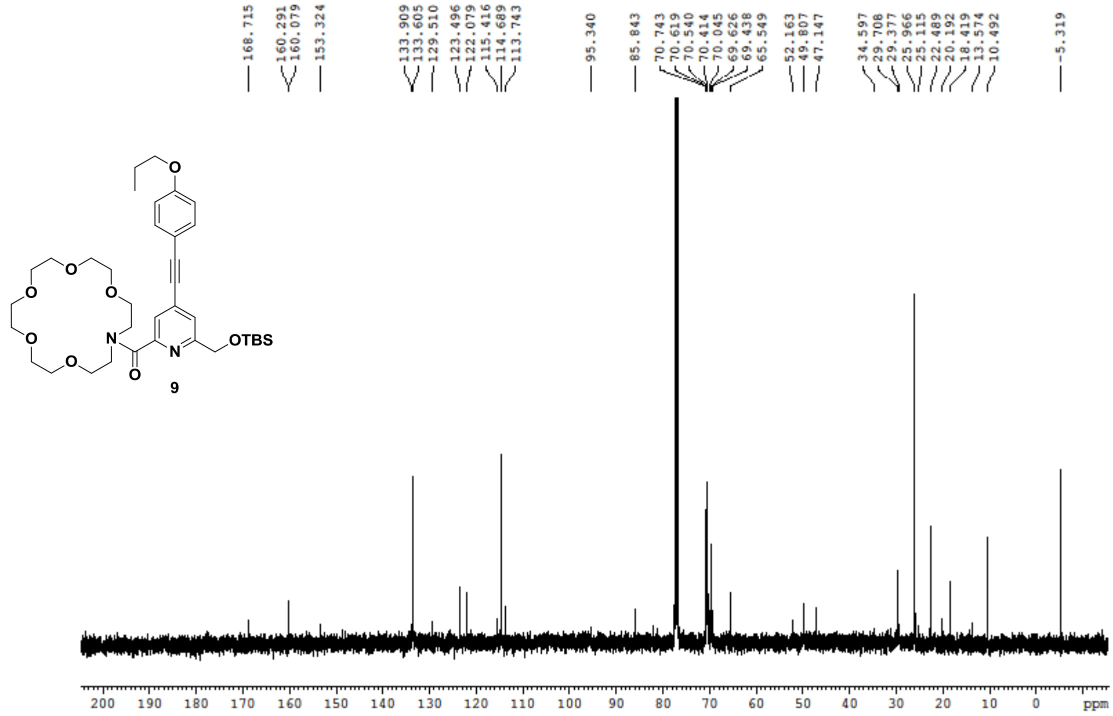

^{1}H NMR and ^{13}C NMR spectra of **10**

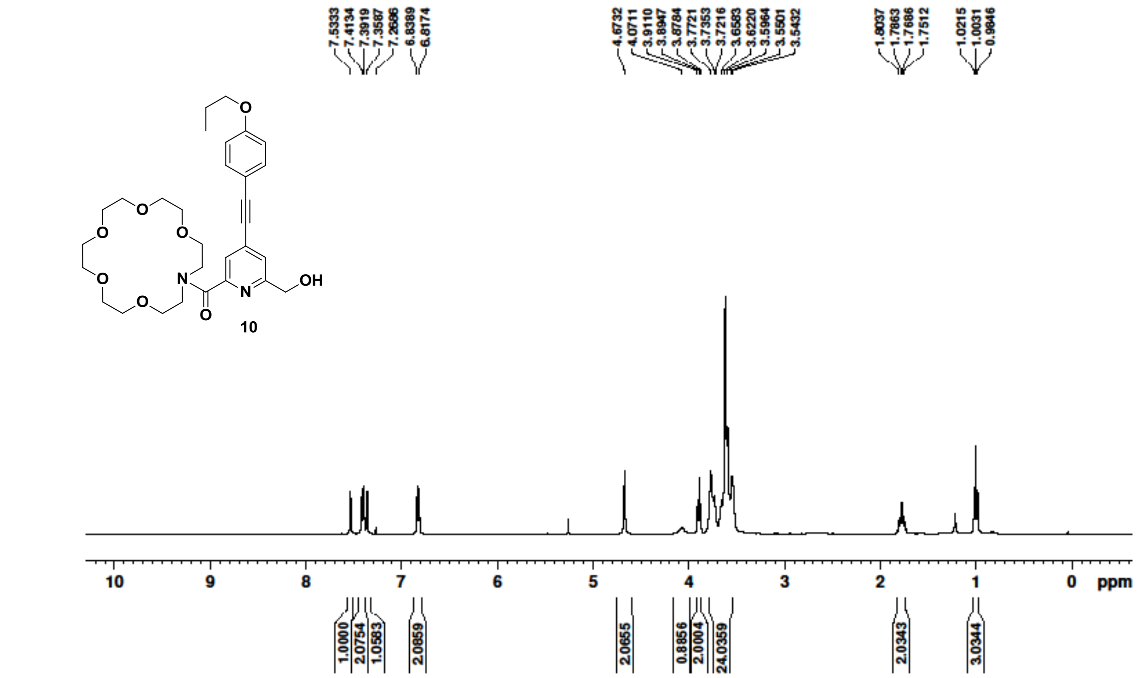

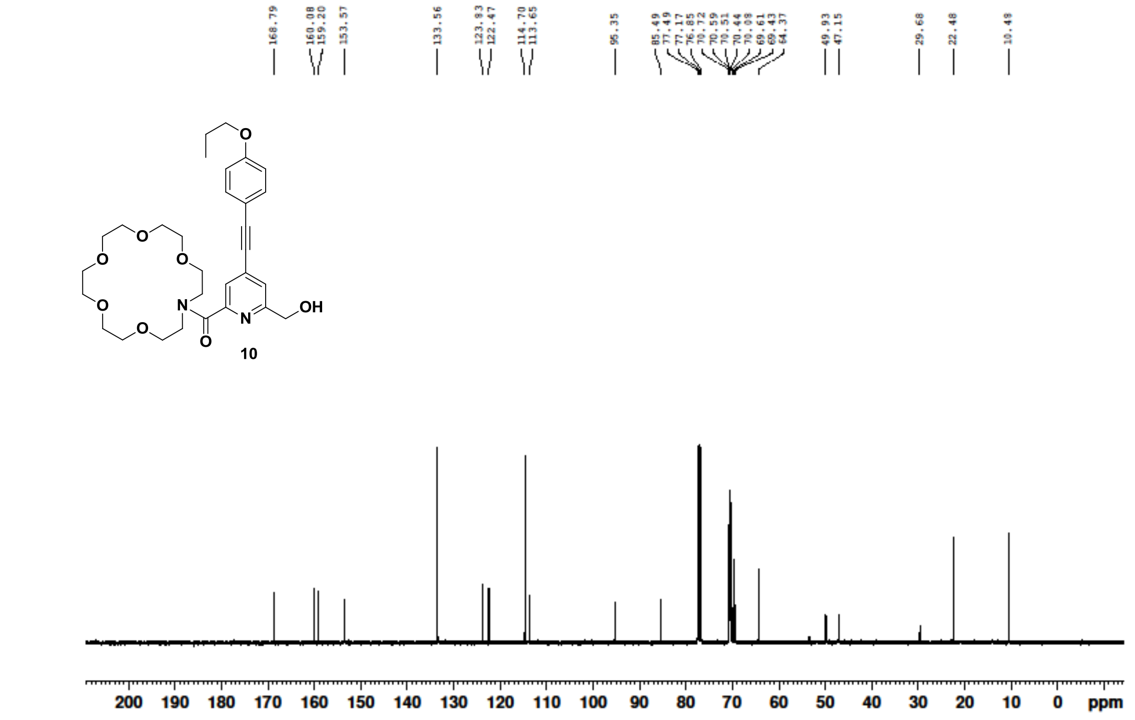

^{1}H NMR and ^{13}C NMR spectra of **11**

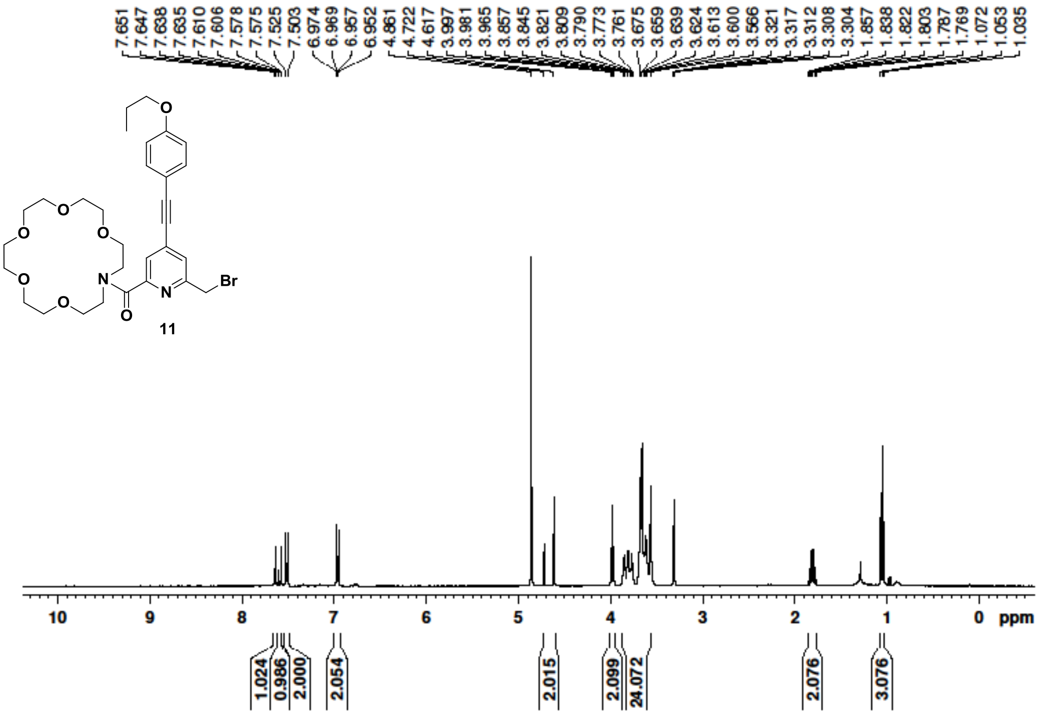

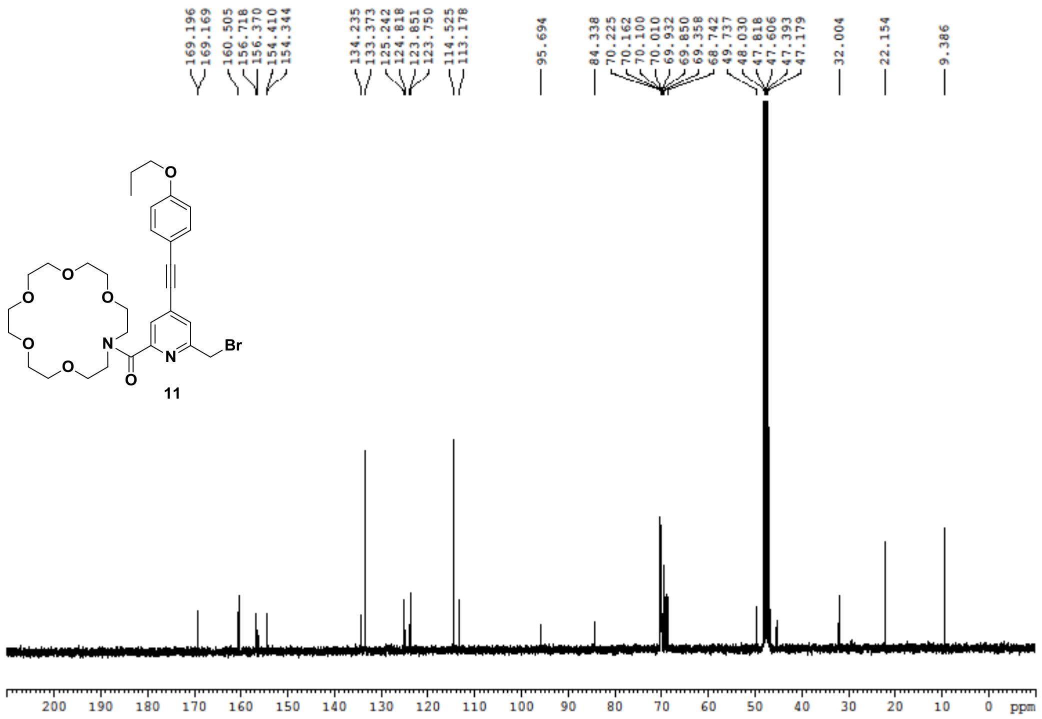

^{1}H NMR and ^{13}C NMR spectra of **L2**

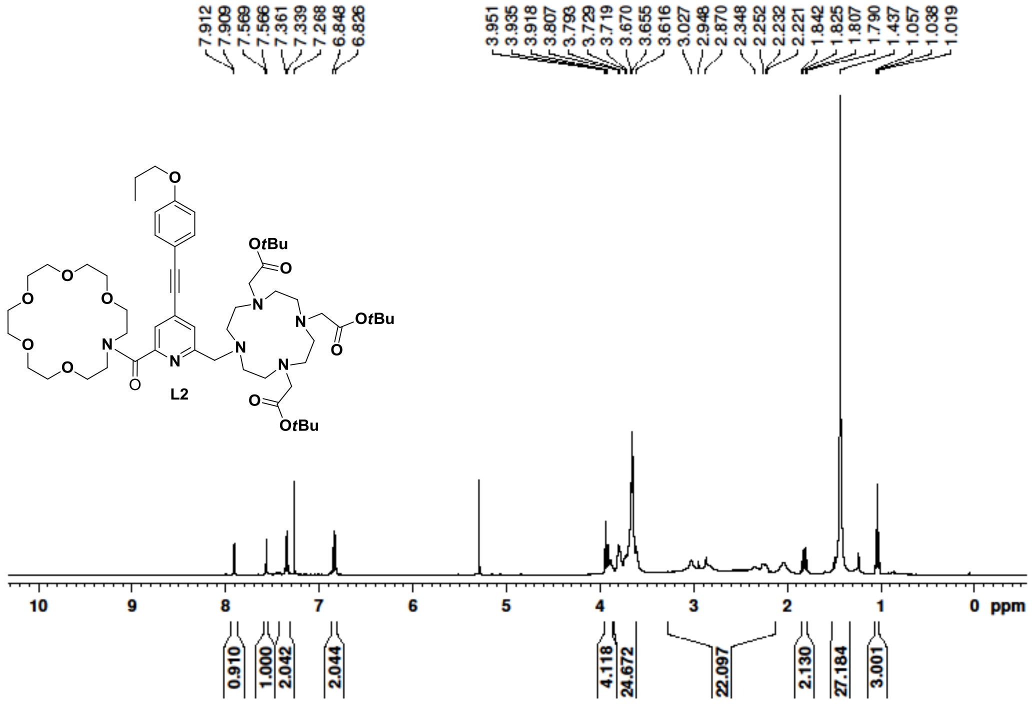

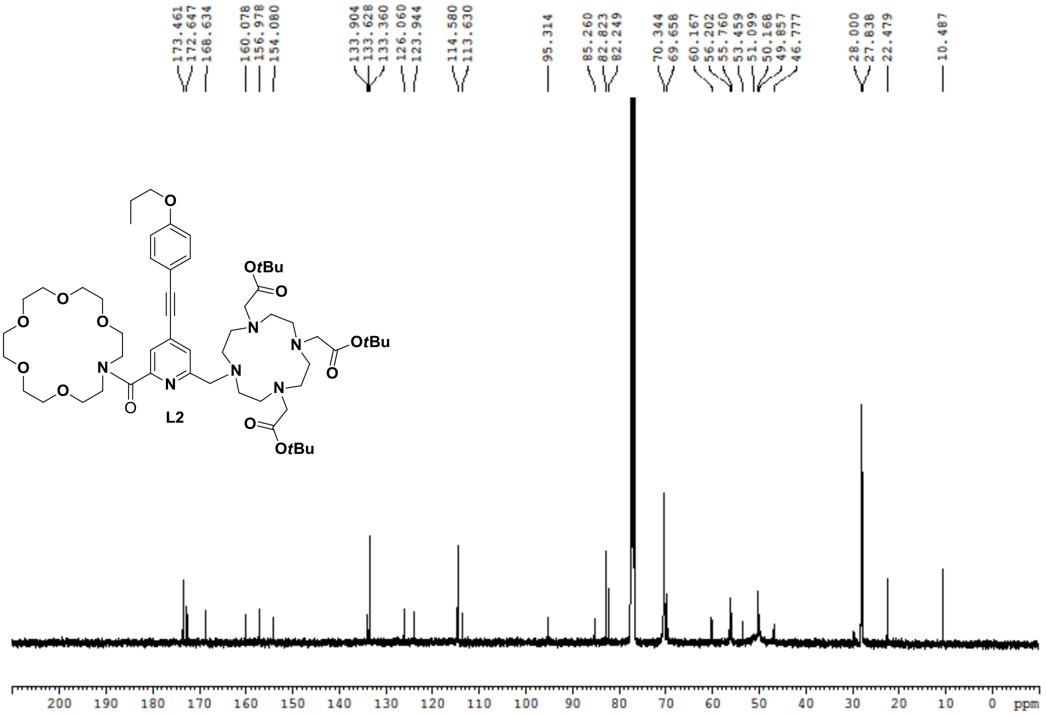

^{1}H NMR and ^{13}C NMR spectra of **L3**

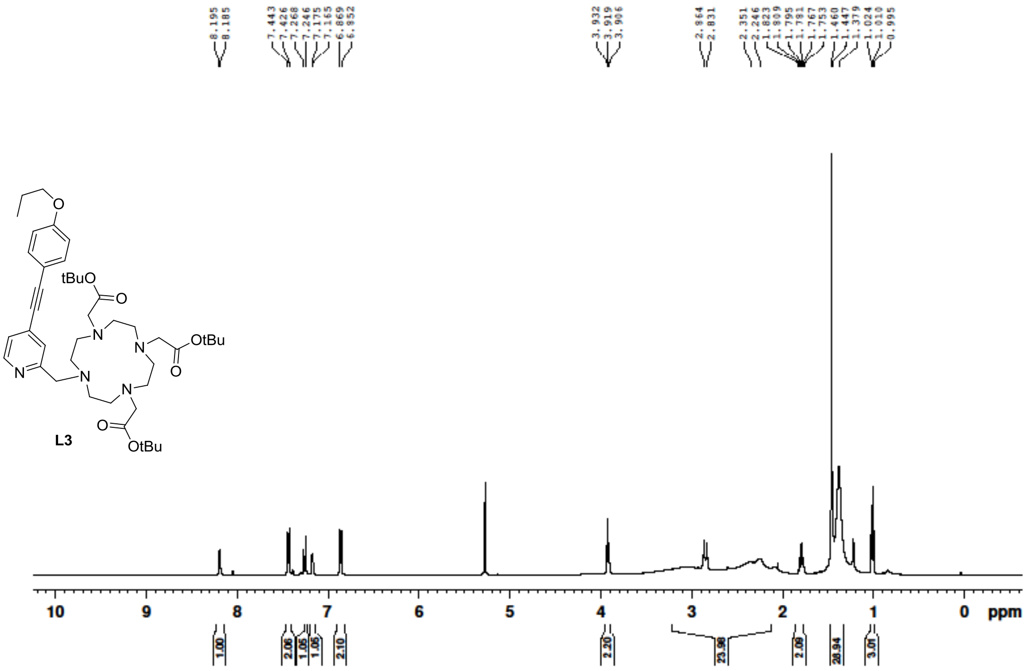

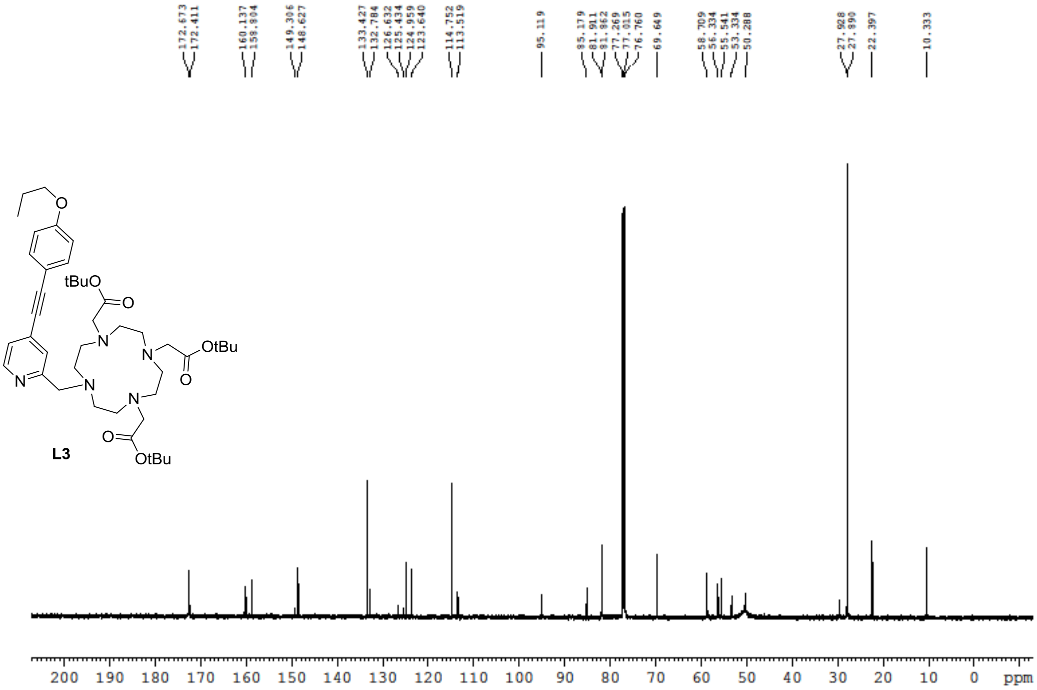

^{1}H NMR and ^{13}C NMR spectra of **L4**

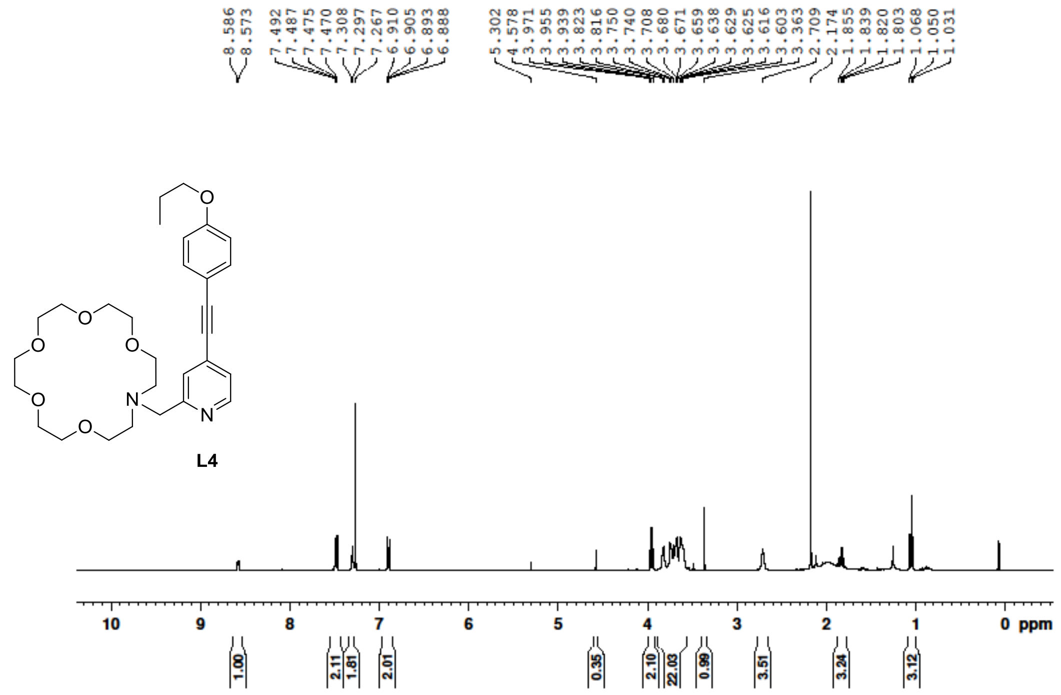

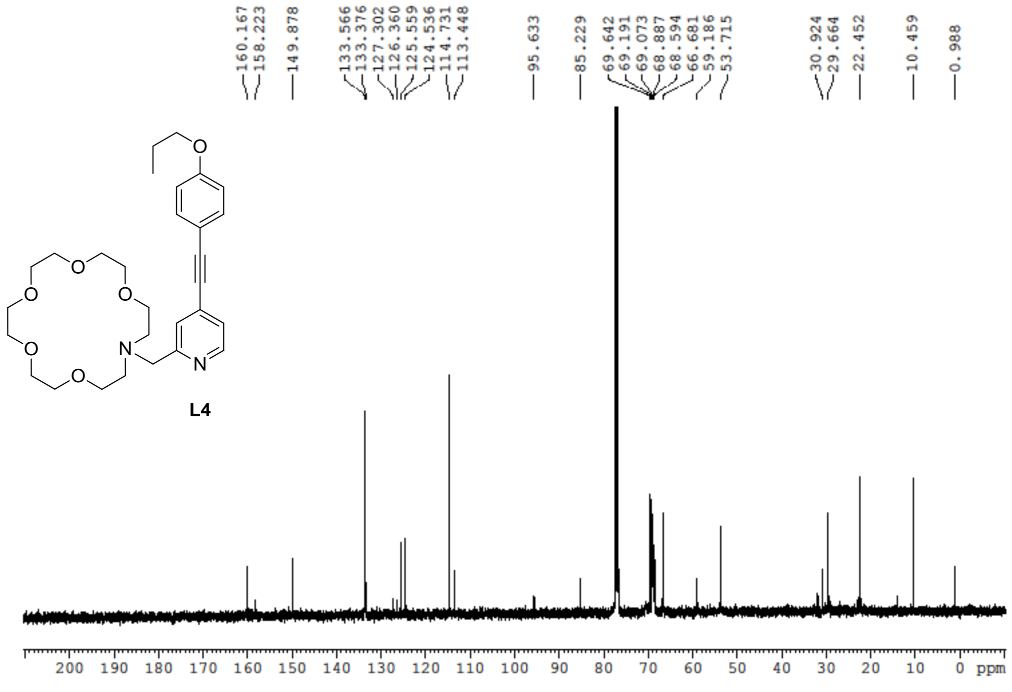

^{1}H NMR and ^{13}C NMR spectra of **L5**

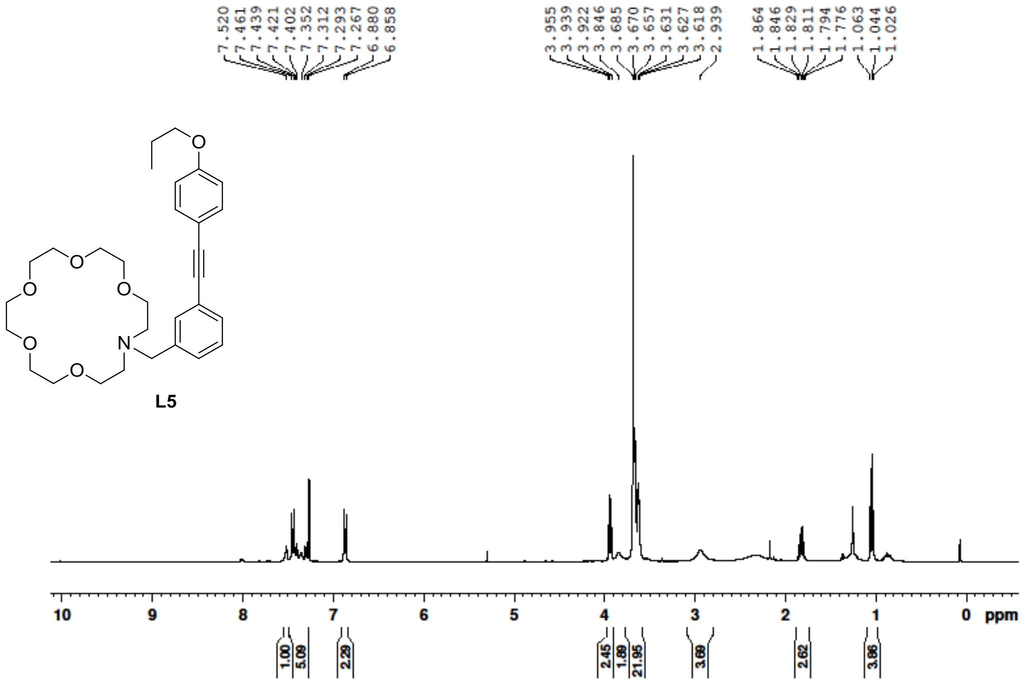

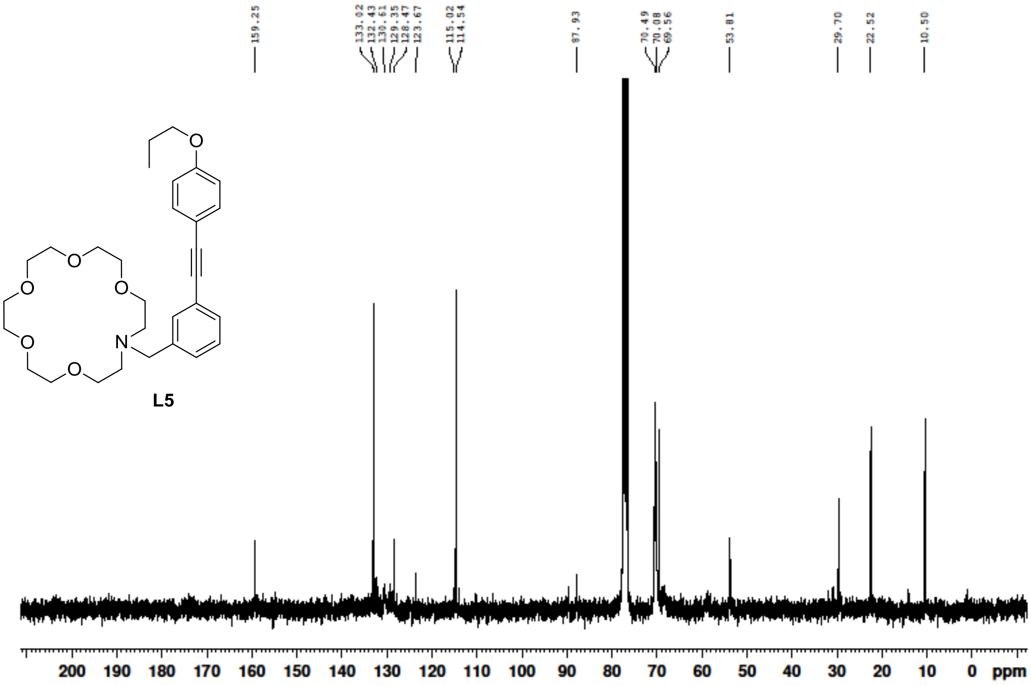

HRMS of **L1**

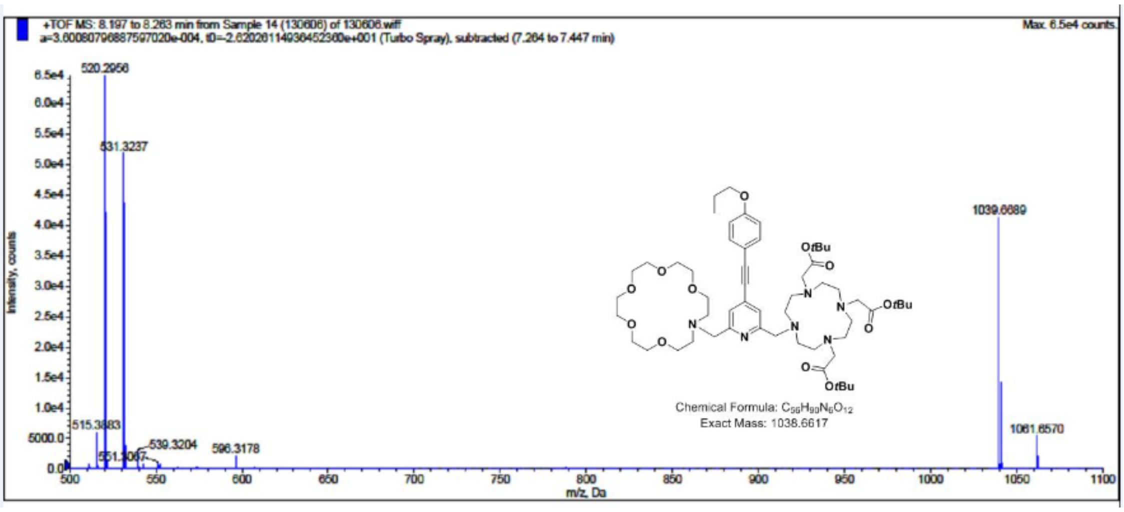

HRMS of **EuL1**

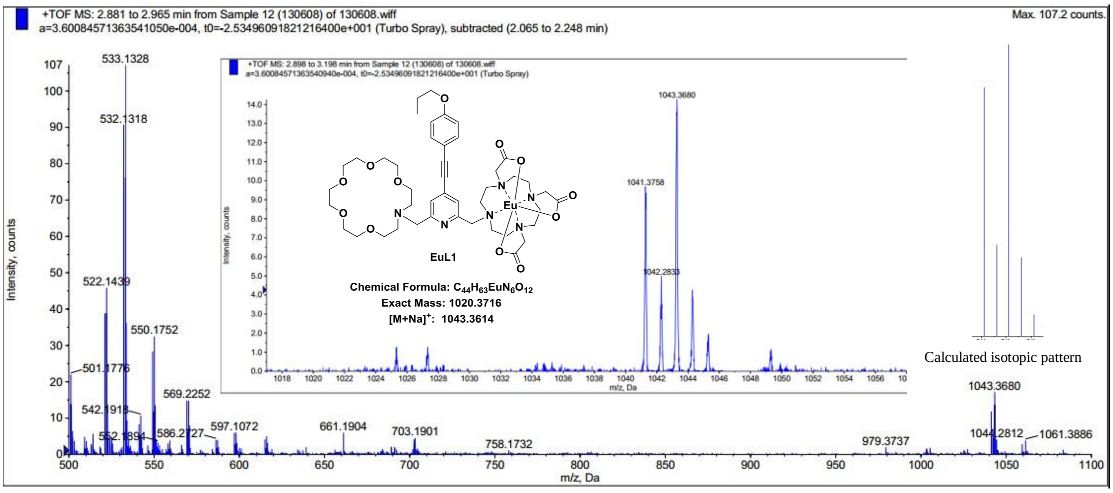

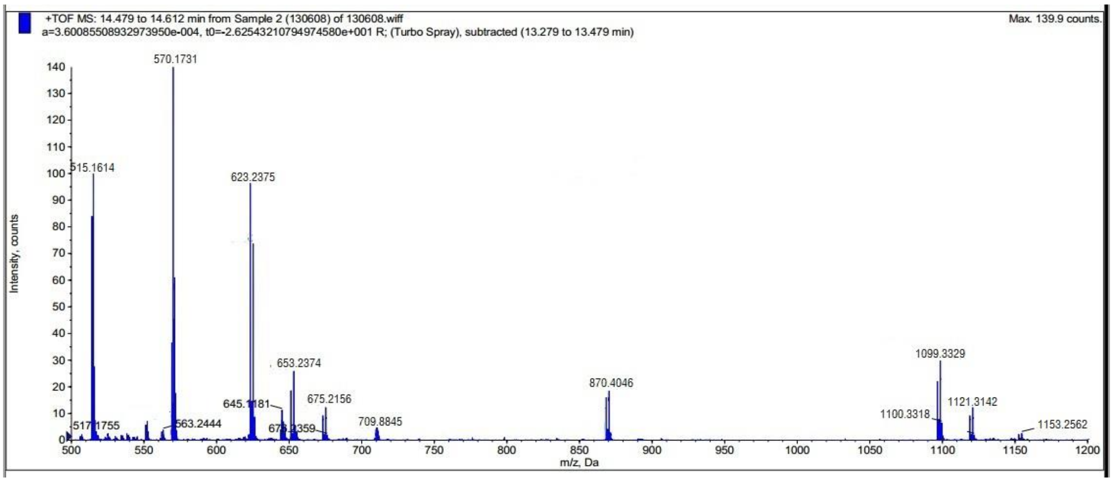

Table S1. Solvent gradient for HPLC

Time /min	0.1% CHOOH in mQ water /%	0.1% CHOOH in CH_3CN /%
0.0	95	5
14.0	60	40
15.0	60	40
20.0	0	100

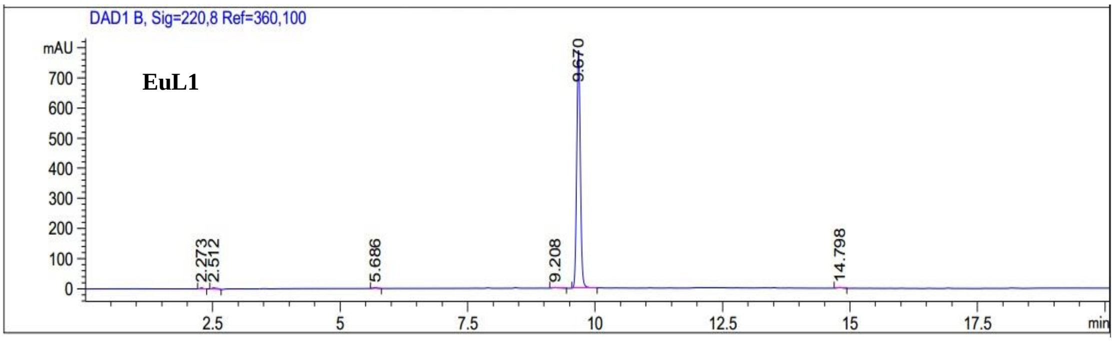

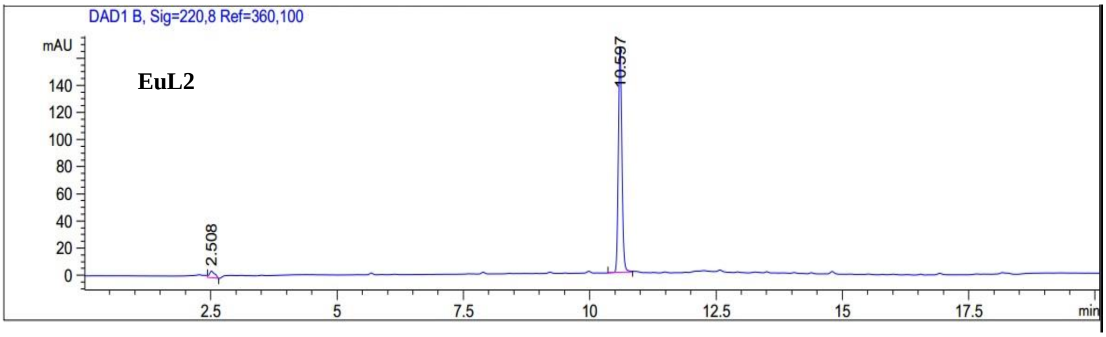

Figure S1 HPLC trace of Eu complexes. Experimental conditions: Agilent ZORBAX SB-C18 Stable Bond Analytical 4.6 X 150 mm 5-micron, 1.0mL/min flow rate, Retention Time: EuL1 in 9.67 min, con.Eu-1 in 10.60 min.

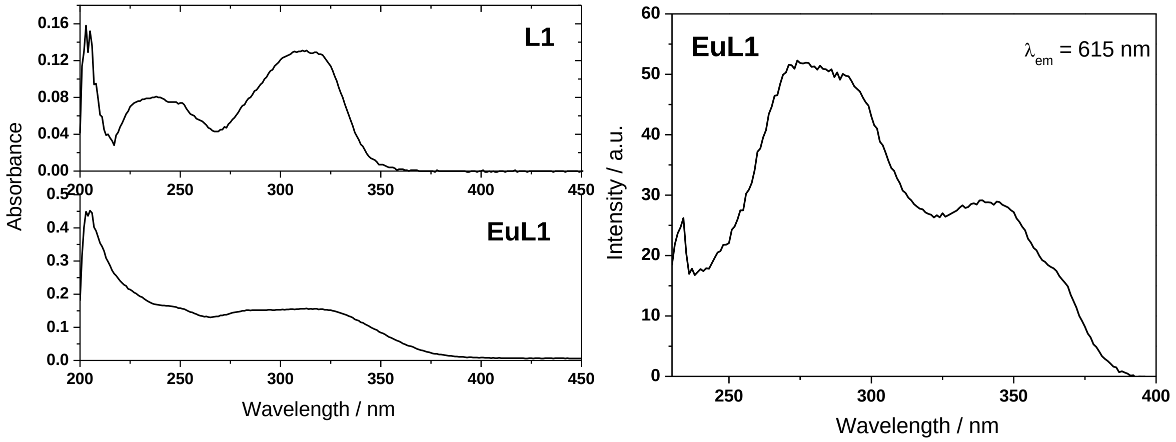

Figure S2 Left: UV-vis absorption spectrum of **L1** (top) and **EuL1** (bottom) in methanol. Right: Excitation spectra of **EuL1**. (H_2O, λ_{em} = 615 nm, 10 μM).

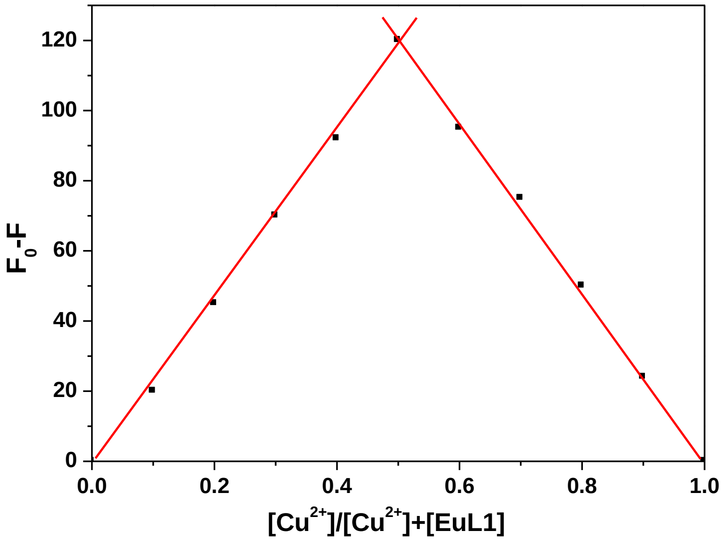

Figure S3 Job's plot of **EuL1** towards Cu^{2+} ions.

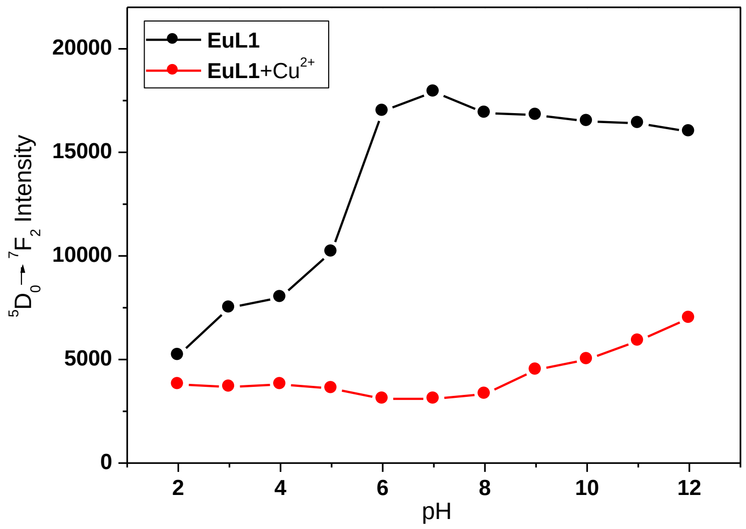

Figure S4 Fluorescence response of **EuL1** at various pH values (2-12) in the absence (black) or presence (red) of Cu^{2+} (10 μM). All spectra were acquired in water with λ_{ex} at 325 nm.

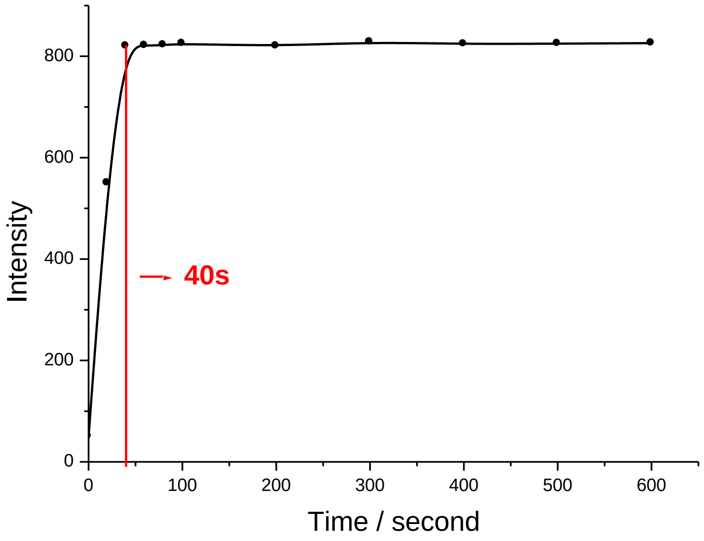

Figure S5 The emission intensity at 615 nm upon addition of H_2S from 0 to 10 min.

Table S2 Luminescence lifetimes (τ/ms), number of inner sphere water molecules (q), quantum yield (φ) of **EuL1** (50 µM in water) alone, with Cu^{2+} ion, and with Cu^{2+} ion and H_2S.

Complex	τ $_{H2O}$	τ $_{D2O}$	q^a	φ (H_2O)^b
**EuL1**	0.61	1.20	0.66	0.5%
**EuL1**+Cu^{2+}	-^{c}	-^{c}	-^{d}	-^{d}
**EuL1**+Cu^{2+}+H_2S	0.34	0.47	0.68	1.1%

^{a} Values of the number of coordinated water molecules, q, (±20%), were determined according to the literature procedures.[5] ^{b} The quantum yields were measured by integrated sphere in aqueous solution. ^{c} The fluorescence signal is too weak to be detected. ^{d} Not determined.

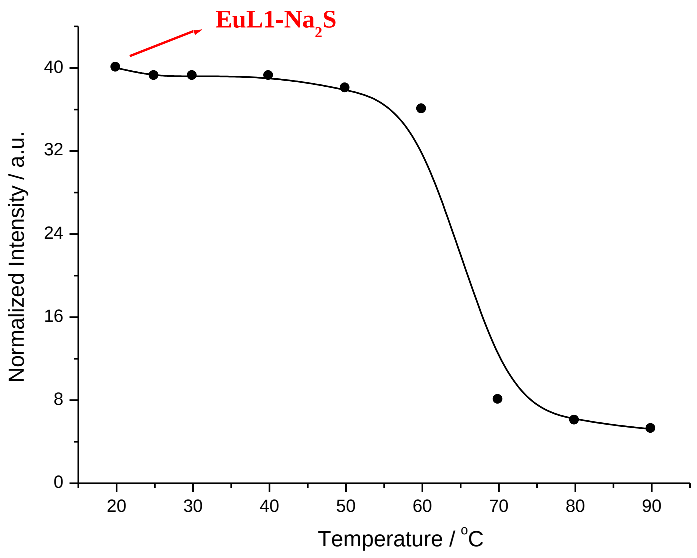

Figure S6 Temperature dependent luminescence study of the **EuL1**-Na_2S complex

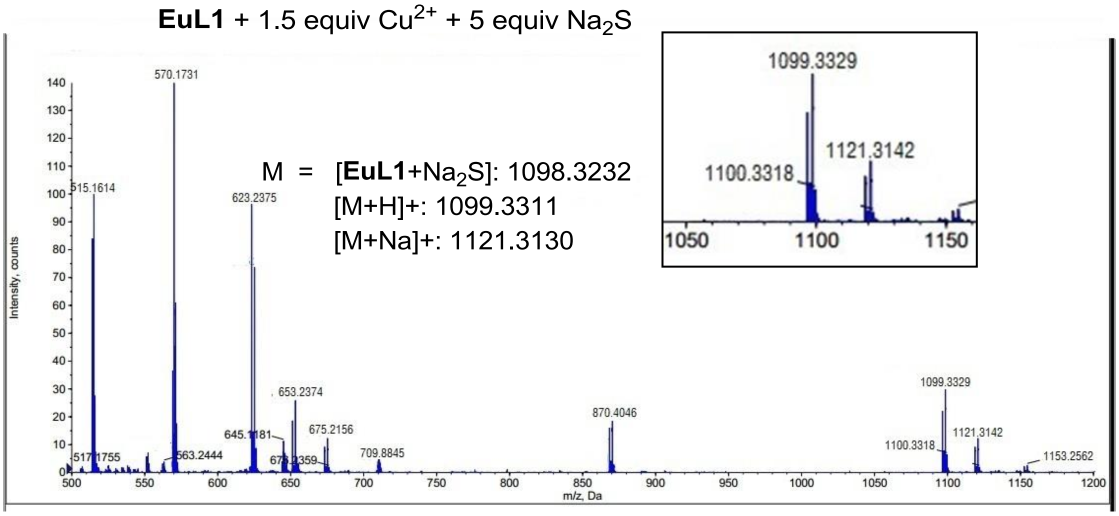

Figure S7 HMRS spectrum of **EuL1** with Cu^{2+} and Na_2S.

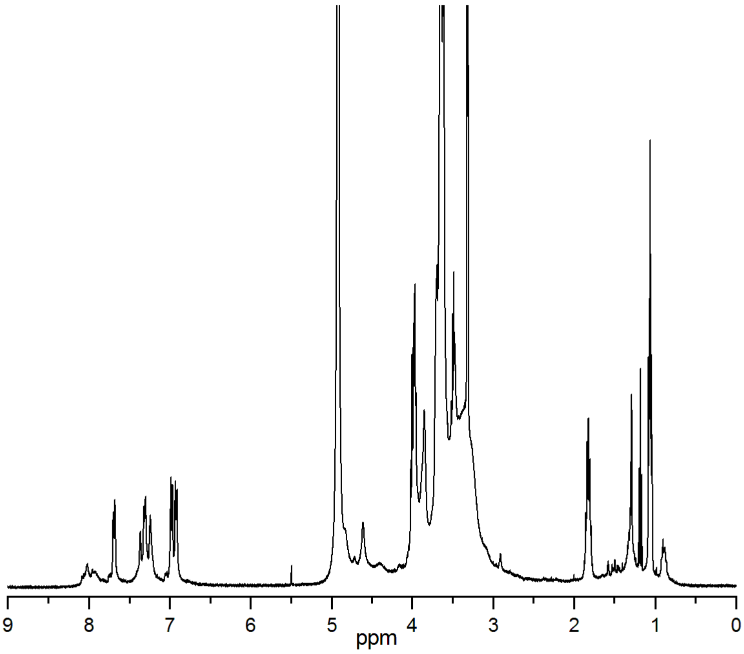

Figure S8 ^{1}H NMR spectrum of **LaL1**

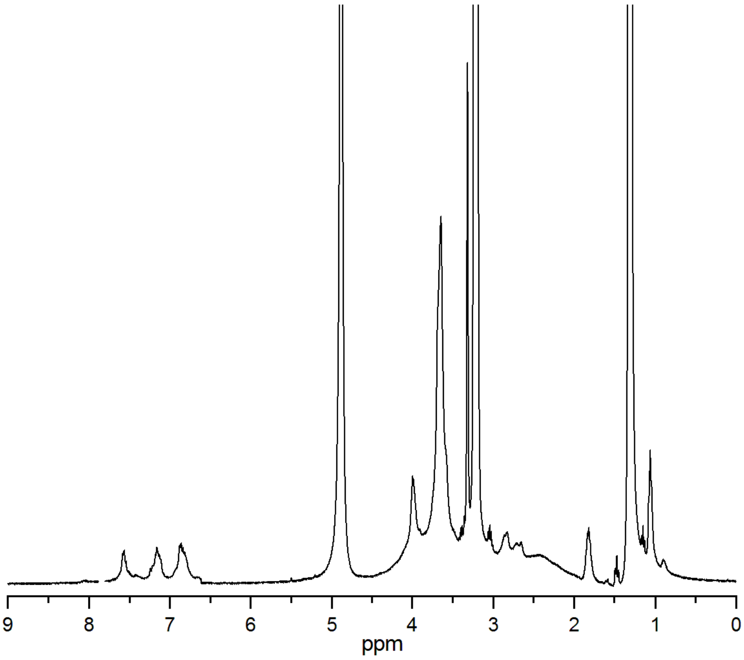

Figure S9 ^{1}H NMR spectrum of **LaL1**+Cu^{2+}

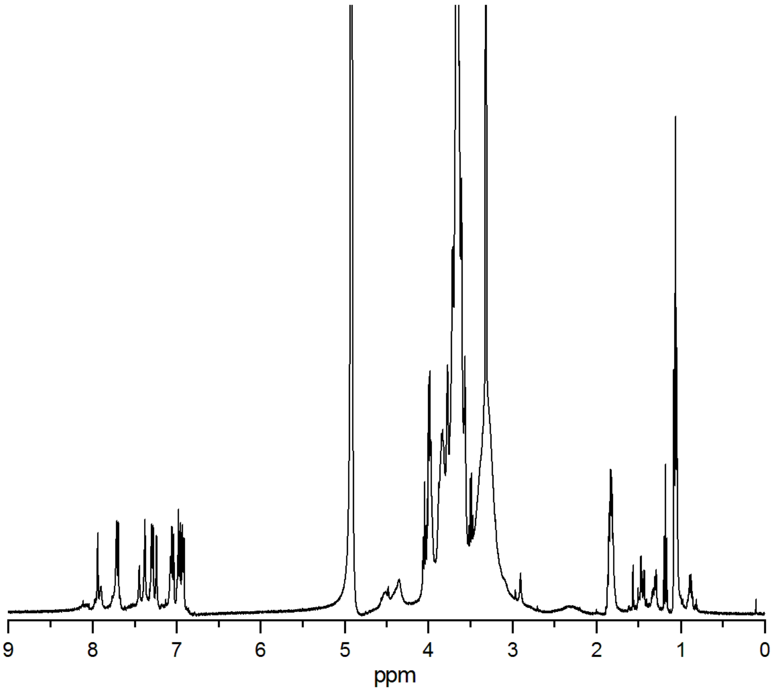

Figure S10 ^{1}H NMR spectrum of **LaL1**+Cu^{2+}+Ha_2S

References

1. (*a*) H. Benesi and J. H. Hildebrand, *J. Am. Chem. Soc.*, 1949, **71**, 2703; (*b*) K. A. Connors, Binding constants: the measurement of molecular complex stability, Wiley, New York, 1987.
2. L. C. Gilday, T. Lang, A. Caballero, P. J. Costa, V. Flix and P. D. Beer, *Angew. Chem. Int. Ed.*, 2013, **52**, 4356.
3. A. Bourdolle, M. Allali, J.-C. Mulatier, B. Le Guennic, J. M. Zwier, P. L. Baldeck , J.-C. G. Bünzli, C. Andraud, L. Lamarque and O. Maury, *Inorg. Chem.*, 2011, **50**, 4987.
4. H.-K. Kong, F. L Chadbourne, G.-L. Law, H. Li, H.-L. Tam, S. L. Cobb, C.-K. Lau, C.-S. Lee and K.-L. Wong *Chem. Commun*., 2011, **47**, 8052.
5. B. Andrew, M. C. Ian, S. D. Rachel, F. Stephen, P. David, P. Louise, S. S. Alvaro and W. Mark, *J. Chem. Soc., Perkin Trans. 1*, 1999, **2**, 493.
